# The predictability of claim-data-based comorbidity-adjusted models could be improved by using medication data

**DOI:** 10.1186/1472-6947-13-128

**Published:** 2013-11-20

**Authors:** Ji Hwan Bang, Soo-Hee Hwang, Eun-Jung Lee, Yoon Kim

**Affiliations:** 1 Department of Health Policy and Management, Seoul National University College of Medicine, 103 Daehak-ro, Jongno-gu, Seoul 110-799, Korea; 2 Division of Infectious Diseases, Seoul National University - Seoul Metropolitan Government Boramae Medical Center, Seoul, Korea; 3 Current address: Health Insurance Review and Assessment Service, Seoul, Korea; 4 Current address: Department of Health Informatics and Management, Chungbuk National University College of Medicine, Cheongju, Korea; 5 Institute of Health Policy and Management, Medical Research Center, Seoul National University, Seoul, Korea

**Keywords:** Severity-of-illness index, Comorbidity, Prescriptions, Drug, Risk-adjustment, Outcome assessment

## Abstract

**Background:**

Recently, claim-data-based comorbidity-adjusted methods such as the Charlson index and the Elixhauser comorbidity measures have been widely used among researchers. At the same time, there have been an increasing number of attempts to improve the predictability of comorbidity-adjusted models. We tried to improve the predictability of models using the Charlson and Elixhauser indices by using medication data; specifically, we used medication data to estimate omitted comorbidities in the claim data.

**Methods:**

We selected twelve major diseases (other than malignancies) that caused large numbers of in-hospital mortalities during 2008 in hospitals with 700 or more beds in South Korea. Then, we constructed prediction models for in-hospital mortality using the Charlson index and Elixhauser comorbidity measures, respectively. Inferring missed comorbidities using medication data, we built enhanced Charlson and Elixhauser comorbidity-measures-based prediction models, which included comorbidities inferred from medication data. We then compared the c-statistics of each model.

**Results:**

247,712 admission cases were enrolled. 55 generic drugs were used to infer 8 out of 17 Charlson comorbidities, and 106 generic drugs were used to infer 14 out of 31 Elixhauser comorbidities. Before the inclusion of comorbidities inferred from medication data, the c-statistics of models using the Charlson index were 0.633-0.882 and those of the Elixhauser index were 0.699-0.917. After the inclusion of comorbidities inferred from medication data, 9 of 12 models using the Charlson index and all of the models using the Elixhauser comorbidity measures were improved in predictability but, the differences were relatively small.

**Conclusion:**

Prediction models using Charlson index or Elixhauser comorbidity measures might be improved by including comorbidities inferred from medication data.

## Background

When reviewing medical records, claim data, such as claim data for payment, is much easier to obtain than clinical data. Thus, in recent years, many researchers have used claim data to predict prognoses for hospitalized patients [1-16]. The most widely adopted methods of predicting patients’ prognoses based on claim data are comorbidity-adjusted models such as the Charlson index and the Elixhauser index [17,18]. However, one problem with comorbidity-adjusted models has been the number of missed comorbidities in claim data. Two separate Canadian studies revealed that 33-48% of comorbidities have been missed in claim data [19,20].

As claim-data-based comorbidity-adjusted models have become increasingly popular, many researchers have tried to find methods of improving the predictability of the comorbidity-adjusted models in order to overcome these limitations [21-25]. Some investigators have made efforts to compensate for problems caused by missed comorbidities in claim data by using drug prescription information. The Chronic Disease Score (CDS) is a prominent example of such efforts [26,27]. However, the performance of the CDS-based models in the prediction of prognoses was inferior to that of diagnosis-based models, including comorbidity-adjusted models [28,29]. The disappointing outcomes of trials using the CDS might be attributed to two flaws of the CDS: first, some drugs included in the CDS can be used for two or more diseases (or conditions), and second, the drugs by themselves may not reflect the severity of diseases (or conditions).

Therefore, it’s fair to assume that an algorithm-based comorbidity inference method combining medication data with the indications of each drug could help to identify missed comorbidities reflecting severity of diseases (or conditions). Our hypothesis is that if missed comorbidities, traced by medication data, were added to comorbidity-adjusted models, the predictabilities of the models might be improved. The purpose of our study is 1) to develop an algorithm-based comorbidity inference method by combining medication data and the indications of each drug to identify missed comorbidities; and 2) to evaluate to what degree the predictive performance of comorbidity-adjusted models can be improved by the addition of drug-inferred comorbidities identified by the algorithm.

## Methods

### Framework of the study

#### Subjects

We obtained inpatient claim data from all South Korean hospitals with more than 700 beds during the year 2008. After excluding malignant diseases, we identified twelve most responsible diagnoses that caused high numbers of in-hospital deaths.

#### Comorbidity-adjusted models for predicting in-hospital mortality

The Charlson index and Elixhauser comorbidity measures were used to build comorbidity-adjusted models for in-hospital mortality (see 'Tools for adjusting severity of comorbidities’ , below, for details), and the predictive performance of each model was evaluated.

#### Inferring missed comorbidities

Using medication, we inferred missed comorbidities from the claim data.

#### Comorbidity-adjusted models for predicting in-hospital mortalities that include drug-inferred comorbidities

We built in-hospital mortality prediction models based on the Charlson and Elixhauser comorbidity measures and at the same time included comorbidities inferred from prescribed medications.

#### Comparison of predictabilities of comorbidity-adjusted models before and after the inclusion of comorbidities inferred from medication data

We compared the predictability of the Charlson and Elixhauser comorbidity-based models that included comorbidities inferred from medication data with the original models of the Charlson and Elixhauser indices, which did not include the comorbidities inferred from medication data.

### Study subjects

The study population consisted of all inpatients in South Korea who were admitted to any of the 66 hospitals identified as having more than 700 beds in 2008. To ensure the validity and stability of the estimates for comorbidity-based mortality prediction, we chose to limit our study to hospitals with more than 700 beds, as they care for more than half of all South Koreans admitted to hospitals. We obtained the claim data of the hospitals included in the study from the HIRA, the organization that reviews all the claims of the entire population of South Korea, regardless of whether they belong to the national health insurance or the medical aid program. For patients who admitted twice or more during the study period, we considered them different cases. If the study subjects had been admitted the previous year, in 2007, we also obtained and utilized the claim data of corresponding cases in 2007 in our construction of the Charlson-index-based models (see 'Tools for adjusting severity of comorbidities’ , below, for details).

First, we identified the most responsible diagnoses (MRDx), which account for 80% of the in-hospital mortalities identified in the claims. MRDx meant the principal diagnoses that were most responsible for the hospitalization. We then excluded the claims with MRDx of malignancies because we could not accurately exclude the cancer patients admitted for palliative care. Regardless of the quality of care, in-hospital mortalities of cancer patients admitted for palliative care will inevitably be much higher than those of cancer patients admitted for active treatment. Thus we did not include the cases with MRDx of malignant diseases (see next paragraph for detail). We grouped MRDx according to the classifications established by the US Agency for Healthcare Research and Quality, but divided acute cerebrovascular diseases into intracranial hemorrhage and ischemic infarct because these two categories are quite different in pathophysiology and therapeutic approach [30].

In Korea, patients can do hospital-shopping unlimitedly. As a result, ratios of cancer patients who undergo active treatment and patients who undergo palliative or terminal care are quietly different even in tertiary care centers. In this condition, in-hospital mortalities of hospitals which have many palliative or terminal cases must be high regardless of the quality of care. We performed this study to develop methodology to rank quality of care of Korean hospitals. If we adapt comorbidity-adjusted models to measure quality of care in cancers, hospitals with many cancer patients for palliative or terminal care must be underrated unjustly. Thus we excluded all the cancer patients.

#### Exclusion criteria

We excluded cases that met any of following criteria:

1. Age ≤ 28 days or ≥ 120 years [31].

2. In-hospital stay > 365 days [31].

3. Transferred cases, including both transferred-in and transferred-out cases: The prognoses of transferred cases might not reflect qualities of care in current hospitals.

4. Most responsible diagnoses that have a wide range of clinical spectrums (ex. sepsis, respiratory failure, intoxications, other lower respiratory diseases, cardiac arrest, ventricular fibrillation): They are not clinically homogeneous categories which are not suitable for a valid and reliable risk-adjusted mortality prediction. This is the first large study which adapted comorbidity adjusted method, thus we decided to include relatively homogenous groups. Moreover, in these cases, the most responsible diagnoses, per se, might be results or complications of treatment rather than POA (present on illness).

### Tools for adjusting the severity of comorbidities

In our study, we adopted the Charlson index with Deyo’s application as well as the Elixhauser comorbidity measures for adjusting the severity of comorbidities. In a comorbidity-adjusted model, comorbidity indicates an associated disease or condition existing before the point of admission and is used interchangeably with Present on Admission (POA). In contrast, a disease or condition that occurred after hospitalization is called a complication or non-POA [19].

#### Charlson index

The Charlson index is the most frequently used method for adjusting the severity of comorbidities in studies based on claim data [17]. In 1987, M.E. Charlson and her colleagues reported 17 comorbidities that influence the prognoses of hospitalized patients, and they gave a “weight” of 1 to 6 to each individual comorbidity, depending on the strength of the treatment outcome: the higher weight, the worse the prognosis tends to be (Additional file 1: Table S1). The sum of weights for comorbid conditions indicates the severity of comorbidities for each individual case. Widely accepted among researchers, the Charlson index has been used to build nation-wide prognoses prediction models in many countries [31-33].

To apply the Charlson index, it is important to distinguish a comorbidity (or POA) from a complication (or non-POA) in associated diagnoses (not MRDx). Deyo’s application has been used more than any other to make this distinction [21]. In Deyo’s application, chronic diseases (or conditions) are classified as POA conditions. In addition, according to Deyo’s application, diseases (or conditions) that are not explicitly chronic must be mentioned in the previous data from at least one year prior to the index admissions in order to be considered as POA conditions; otherwise, they are regarded as non-POA conditions (Additional file 1: Table S1).

#### Elixhauser comorbidity index

Elixhauser et al. found 31 comorbidities that influence the prognoses of hospitalized patients (when hypertension is divided between uncomplicated hypertension and complicated hypertension) (Additional file 1: Table S2) [18]. In the Elixhauser comorbidity index, existence or non-existence of specific comorbidities is used to adjust the severity of comorbidities. The Elixhauser comorbidity index is generally accepted as similar or superior to the Charlson index in its prediction of prognoses [34-37]. However, Elixhauser-index-based models could be unstable due to the high number of independent variables in the model.

In order to discriminate between POA conditions and non-POA conditions, the Diagnosis-related Group (DRG) screening method was used in the Elixhauser comorbidity measures. The DRG screening method classified secondary diagnoses as non-POA when the method determined that secondary diagnoses belonged to the same disease category as the MRDx and/or resulted from MRDx; otherwise, they were considered POA conditions.

Because the International Classification of Diseases-10th Edition (ICD-10) has been used for diagnostic coding in South Korea since 1995, Quan’s algorithm was applied to convert ICD-10 diagnoses to the ICD-9-CM diagnoses on which the Charlson index and Elixhauser comorbidity index were originally based [38].

### Inferring missed comorbidities

In order to infer missed comorbidities in the claim data, we used medication data. Specifically, drugs had to meet all of following criteria to be used for the inference of missed comorbidities: 1) prescribed one or more times during hospitalization; 2) used for treatment of diseases (or conditions) included in Charlson index and/or Elixhauser comorbidity measures; and 3) used exclusively for one disease entity.

We excluded drugs that met any of following criteria: 1) Drugs that can be used for more than one disease entity under the prescription guidance of the HIRA [39]; 2) Drugs possibly used in relatively mild abnormal conditions including topical agents; 3) Antineoplastics; 4) Parenteral drugs. For example, paroxetine, one of the most frequently prescribed antidepressants, was excluded because it could be used to treat other conditions such as obsessive-compulsive disorder. In the Elixhauser comorbidity measures, uncomplicated hypertension and complicated hypertension are classified as different entities. If a patient was prescribed an antihypertensive, we could not determine whether he or she had uncomplicated hypertension or complicated hypertension. Thus, we excluded antihypertensives. Similarly, in both the Charlson index and the Elixhauser comorbidity index, uncomplicated diabetes is distinguished from complicated diabetes. Thus, we excluded antidiabetic drugs. Antineoplastic drugs were excluded because they could be used not only for cancer patients, but also for patients with connective tissue diseases and transplantation patients. Parenteral drugs were excluded because many parenteral drugs were used to treat conditions that appeared after admission.

Finally, to eliminate non-POA conditions from the inferred comorbidities, the DRG screening method of the Elixhauser comorbidity measure was used (see 'Tools for adjusting severity of comorbidities’ for details) [18].

### Building prediction models for in-hospital mortalities

In this study, we built prediction models for in-hospital mortalities using multiple logistic regression analyses. The independent variables of each model were: age, sex, status of health insurance (the National Health Insurance beneficiaries or Medical Aids beneficiaries), admission category (emergency admission or not), operative status (operated on or not), and adjusted severity of comorbidities measured by the Charlson index or Elixhauser comorbidity measures. Following our application of the aforementioned methods, we built four models for each disease in the study, as indicated below.

1. Charlson models: Charlson-index-based comorbidity-adjusted models that use only comorbidities described in the claim data.

2. Elixhauser models: Elixhauser-comorbidity-measure-based models that use only comorbidities described in the claim data.

3. Enhanced Charlson models: Charlson-index-based comorbidity-adjusted models that use both comorbidities described in the claim data and inferred from medication data.

4. Enhanced Elixhauser models: Elixhauser-comorbidity-measure-based comorbidity-adjusted models that use both comorbidities described in the claim data and inferred from medication data.

### Statistic validation of the models

We calculated the c-statistic for each model and compared them to measure whether the addition of drug-inferred comorbidities could improve the predictive power of the Charlson and Elixhauser comorbidity indices. The c-statistic is an indicator of predictability. If the c-statistic = 0.5, it means the predictability of the model is 0%. If the c-statistic = 1.0, it means the predictability of the model is 100%. Additionally, we carried out Hosmer-Lemeshow Goodness-of-Fit tests to examine the fitness of the models. We performed bootstrapping method to calculate a 95% confidence interval of the c-statistics of each model.

Changes of Charlson index scores in original and enhanced models were compared by Wilcoxon signed rank test.

All the statistical analyses were performed by SAS v9.2 (SAS Institute Inc., Cary, NC, USA).

### Ethics statement

According to the policy of our institution (Seoul National University, College of Medicine), researches requested by the government to the public interests are to be exempted from approval by the Institutional Review Board (IRB). Our study was proposed by the governmental organization of Korea (The HIRA). Therefore this study was accomplished without ethical review of the IRB.

## Results

### General characteristics of the study population

Total 706,321 admissions with 33 MRDx were account for 80% of in-hospital mortalities during study period. Excluding the cased with MRDx of wide range of clinical spectrums and malignancies, finally 12 MRDx with 247,712 admission cases were included in this study (Table 1). The overall in-hospital mortality rate was 6.8% (16,962 cases). In the 12 MRDX, intracranial hemorrhage was the most frequent MRDx of in-hospital mortalities, followed by pneumonia, ischemic infarct, acute myocardial infarction, and non-alcoholic liver disease. The majority of study cases were adults (median age: 61 years, interquartile range: 47-71), except cases of pneumonia (median age: 4 years, interquartile range: 1-63), and 59.1% of the cases (146,484 cases) were male.

**Table 1:** **Most responsible diagnoses which were account for 80**% **of in-hospital mortalities during study period**

**MRDx**^**1**^	**No. of death**	**No. of admission**	**Male (%)**	**Mortality rate (%)**	**Age, years (IQR**^**2**^**)**	**LOS**^**3**^**, days (IQR**^**2**^**)**
Liver cancer	3,657	56,433	78.4	6.5	60 (52-67)	6 (4-11)
Lung cancer	3,591	66,116	73.5	5.4	65 (57-71)	6 (4-11)
Stomach cancer	2,421	75,545	68.2	3.2	59 (50-68)	5 (3-11)
Sepsis	1,867	7,090	54.4	26.3	52 (1-72)	7 (4-15)
ICH^4*^	1,661	13,340	50.6	12.5	60 (49-70)	18 (8-30)
Pneumonia*	1,342	48,587	56.7	2.8	4 (1-63)	6 (5-9)
Leukemia	1,015	16,796	58.5	6.0	39 (13-58)	8 (3-24)
Pancreatic cancer	967	12,509	60.2	7.7	64 (55-70)	6 (2-13)
Other gastrointestinal cancer	937	13,863	52.3	6.8	64 (55-71)	7 (4-16)
Colon cancer	913	49,480	57.7	1.9	62 (53-69)	4 (3-7)
Ischemic infarct*	882	30,349	56.3	2.9	68 (58-75)	9 (6-15)
AMI^5*^	855	13,912	68.7	6.1	65 (54-73)	7 (5-9)
Non-alcoholic liver disease*	726	15,246	58.6	4.8	53 (44-65)	8 (4-14)
Intracranial injury*	620	9,676	69.5	6.4	53 (35-68)	10 (4-19)
Non-Hodgkin’s lymphoma	583	16,747	60.5	3.5	56 (43-67)	5 (2-11)
Rectal and anal cancer	581	34,853	63.2	1.7	61 (53-69)	5 (4-9)
Respiratory failure	571	2,317	60.0	24.6	64 (8-75)	9 (5-20)
Breast cancer	563	48,483	0.42	1.2	48 (42-56)	3 (2-8)
CRF^6*^	553	17,921	54.6	3.1	59 (47-69)	6 (4-16)
Intoxication	433	4,751	57.0	9.5	50 (37-65)	3 (2-7)
COPD^7*^	407	12,162	64.4	3.3	68 (56-76)	8 (5-12)
Gastrointestinal bleeding	406	15,039	72.1	2.7	58 (46-70)	6 (3-9)
Alcoholic liver disease*	401	7,983	88.0	5.0	51 (45-59)	8 (5-14)
Aspiration pneumonia*	398	2,573	68.7	15.5	72 (60-80)	14 (7-26)
CHF^8*^	394	7,892	40.0	5.0	74 (66-80)	8 (5-12)
Other lower respiratory disease	363	8,818	58.4	4.1	61 (50-71)	6 (3-10)
ARF^9^	344	3,406	55.2	10.1	67 (50-76)	9 (5-16)
Esophageal cancer	342	7,873	92.7	4.3	65 (58-70)	6 (3-13)
Head and neck cancer	297	11,669	80.1	2.6	59 (50-68)	6 (2-12)
Coronary atherosclersis*	287	68,071	59.2	0.4	63 (54-70)	3 (2-5)
Secondary malignacy	282	6,055	52.6	4.66	60 (50-75)	8 (4-16)
Cardiac arrest	278	766	64.8	36.3	59 (47-70)	7 (2-20)
**Sum**	**28,937**	**706,321**	**59.7**	**4.1**	**60 (48–69)**	**5 (3-11)**

1 Most responsible diagnoses, 2 Interquartile range, 3 Length of stay, 4 Intracranial hemorrhage, 5 Acute myocardial infarction, 6 Chronic renal failure, 7 Chronic obstructive pulmonary disease, 8 Congestive heart failure, 9 Acute renal failure.

*Cases with most responsible diagnoses marked with asterisks were selected for final analysis.

### Charlson models and enhanced Charlson models

Among the 965 drugs (by generic name) prescribed in the study cases, 55 drugs were selected to infer missed comorbidities of the Charlson index in the claim data after the author’s (JH Bang) review of the patients’ medication data and HIRA’s prescriptions guidelines (Additional file 1: Table S3).

Out of the 17 comorbidity conditions on the Charlson index, the following eight conditions could be inferred from medication data: congestive heart failure, peripheral vascular disease, cerebrovascular disease, dementia, chronic pulmonary disease, connective tissue disease and rheumatic disease, renal disease, and AIDS. Of the 26,113 inferred and/or documented cases of peripheral vascular disease, 74.3% (19,406 cases) were detected only by inferring from medication data. With regard to other medical conditions, the percentage of cases inferred by medication data were as follows: congestive heart failure (41.5%), chronic pulmonary disease (39.0%), renal disease (7.3%), cerebrovascular disease (5.6%), AIDS (3.8%), connective tissue disease/rheumatic disease (1.5%), and dementia (1.2%) (Table 2). Changes of Charlson index scores in original and enhanced models were statistically significant in all the 12 pairs (*P* < 0.01 in all the pairs, data not shown).

**Table 2:** Additional Charlson index comorbidities inferred from drug prescribing information (N = 247,712)

**Comorbidities**	**Claim data**^**1**^**(A)**	**Prescribing information**^**2**^**(B)**	**Claim data ∩ Prescribing information**^**3**^**(A∩B)**	**Fraction of additional comorbidities inferred by prescribing information {(A∪B)-A}/(A∪B)**
Peripheral vascular disease	6,707	21,858	2,452	74.3%
Congestive heart failure	4,531	4,159	946	41.5%
Chronic pulmonary disease	30,656	36,129	16,517	39.0%
Renal disease	8,298	1,054	400	7.3%
Cerebrovascular disease	8,196	638	153	5.6%
AIDS	51	10	8	3.8%
Connective tissue disease/rheumatic disease	2,727	114	72	1.5%
Dementia	3,811	261	216	1.2%

1 No. of cases of each Charlson index comorbidity identified from claim data, 2 No. of cases each Charlson index comorbidity inferred from drug prescribing information, 3 No. cases of each Charlson index comorbidity identified (or inferred) both claim data and drug prescribing information.

Comparing the predictive power of the original Charlson model and the enhanced models using c-statistics, the enhanced models were slightly superior to the original in all but three MRDx categories (See Additional file 1: Tables S5-S28 to review the model coefficients). Improvements of c-statistics, however, were relatively small and c-statistics of the enhanced models were within 95% confidence intervals of the original Charlson models (Table 3). The c-statistics of the original models ranged from 0.633 (congestive heart failure) to 0.882 (pneumonia) while those of the enhanced models ranged from 0.641 (congestive heart failure) to 0.884 (pneumonia). In models for intracranial injury and aspiration pneumonia, the c-statistics of the original and enhanced models were equal. In intracranial hemorrhage, the c-statistic of the original model was slightly higher than that of the enhanced model (0.655 and 0.654, respectively).

**Table 3:** **Results of original Charlson models**^**1 **^**and enhanced Charlson**^**2 **^**models**

**MRDx**^**3**^	**c statistics**		**Hosmer-Lemeshow test**	
	**Charlson**^**4**^	**Enhanced**^**5**^	**Charlson**^**4**^	**Enhanced**^**5**^
	**(95%CI**^**6**^**)**	**(95%CI**^**6**^**)**	**chi-square (*P*)**	**chi-square (*P*)**
ICH^7^	0.655	0.654	6.2 (0.63)	10.0 (0.27)
	(0.642-0.669)	(0.641-0.667)		
Pneumonia	0.882	0.884	28.9 (<0.01)	26.7 (0.00)
	(0.876-0.888)	(0.878-0.890)		
Ischemic infarct	0.715	0.716	10.5 (0.23)	12.7 (0.12)
	(0.698-0.750)	(0.699-0.733)		
AMI^8^	0.766	0.770	9.2 (0.32)	10.9 (0.21)
	(0.750-0.782)	(0.754-0.786)		
Non-alcoholic liver disease	0.740	0.750	30.3 (<0.01)	32.2 (<0.01)
	(0.724-0.756)	(0.734-0.766)		
Intracranial injury	0.724	0.724	18.9 (0.02)	16.3 (0.04)
	(0.705-0.743)	(0.705-0.747)		
CRF^9^	0.752	0.756	5.6 (0.69)	5.0 (0.76)
	(0.733-0.771)	(0.737-0.775)		
COPD^10^	0.719	0.726	3.6 (0.89)	2.8 (0.95)
	(0.696-0.742)	(0.704-0.748)		
Alcoholic liver disease	0.696	0.708	30.5 (<0.01)	27.1 (<0.01)
	(0.673-0.719)	(0.685-0.731)		
Aspiration pneumonia	0.658	0.658	12.4 (0.13)	8.8 (0.36)
	(0.631-0.685)	(0.631-0.685)		
CHF^11^	0.633	0.641	6.2 (0.62)	4.7 (0.80)
	(0.604-0.662)	(0.613-0.669)		
Coronary atherosclerosis	0.847	0.861	16.3 (0.04)	21.0 (0.01)
	(0.827-0.867)	(0.842-0.880)		

1 & 2 Multiple logistic regression models for predicting in-hospital mortalities composed of age + sex + status of health insurance + admission category (emergent or not) + operation (yes or no) + Charlson index score, before (Charlson models) and after (enhanced Charlson models) adding comorbidities inferred by drug prescription information, 3 Most responsive diagnoses, 4 Charlson models, 5 Enhanced Charlson models, 6 95% confidence interval calculated by bootstrapping, 7 Intracranial hemorrhage, 8 Acute myocardial infarction, 9 Chronic renal failure, 10 Chronic obstructive pulmonary disease, 11 Congestive heart failure.

### Elixhauser models and enhanced Elixhauser models

After the author’s (JH Bang) review of the patients’ medication data and HIRA’s prescriptions guidelines, 106 drugs were selected to infer missed comorbidities on the Elixhauser index in the claim data (Additional file 1: Table S4). Out of 31 comorbidity conditions on the Elixhauser index, the following fourteen conditions could be inferred from medication data: congestive heart failure, cardiac arrhythmia, peripheral vascular disorders, other neurological disorders, chronic pulmonary disease, hypothyroidism, renal failure, liver disease, AIDS, rheumatoid arthritis and collagen vascular diseases, weight loss, deficiency anemia, psychoses, and depression. As with the enhanced Charlson models, in the 26,113 inferred and/or documented cases of peripheral vascular disease, large numbers of cases (19,406 cases, or 74.3%) were detected only by inferring from medication data. Peripheral vascular disease was followed by psychosis (65.0%), chronic pulmonary disease (43.0%), congestive heart failure (33.6%), deficiency anemia (32.5%), depression (21.7%), other neurological disorders (16.7%), hypothyroidism (14.2%), renal failure (11.7%), weight loss (5.4%), AIDS (5.4%), cardiac arrhythmia (3.1%), rheumatoid arthritis/collagen vascular diseases (2.0%), liver disease (0.3%) (Table 4).

**Table 4:** Additional Elixhauser comorbidity measures inferred from drug prescribing information (N = 247,712)

**Comorbidities**	**Claim data**^**1**^**(A)**	**Prescribing information**^**2**^**(B)**	**Claim data ∩ Prescribing information**^**3**^**(A∩B)**	**Fraction of additional comorbidities inferred by prescribing information {(A∪B)-A}/(A∪B)**
Peripheral vascular disorders	6,707	21,858	2,452	74.3%
Psychoses	1,719	4,614	1,420	65.0%
Chronic pulmonary disease	26,038	36,129	16,517	43.0%
Congestive heart failure	5,423	4,159	1,419	33.6%
Deficiency anemia	12,518	12,949	6,927	32.5%
Depression	8,267	4,420	2,134	21.7%
Other neurological disorders	7,037	2,900	1,491	16.7%
Hypothyroidism	5,346	2,792	1,909	14.2%
Renal failure	4,952	1,054	400	11.7%
Weight loss	7,445	556	130	5.4%
AIDS	35	10	8	5.4%
Cardiac arrhythmia	10,310	975	646	3.1%
Rheumatoid arthritis/ collagen vascular diseases	3,530	144	73	2.0%
Liver disease	21,051	255	187	0.3%

1 No. of cases of each Elixhauser comorbidity identified from claim data, 2 No. of cases each Elixhauser comorbidity inferred from drug prescribing information, 3 No. cases of each Elixhauser comorbidity identified (or inferred) both claim data and drug prescribing information.

The c-statistics of the original Elixhauser model ranged from 0.699 (congestive heart failure) to 0.917 (pneumonia) while those of the enhanced models ranged from 0.707 (congestive heart failure) to 0.920 (pneumonia) (Table 5, see Additional file 1: Tables S29-S52 to review the model coefficients). Comparing the predictive power of the original and enhanced Elixhauser models using c-statistics, the enhanced models were slightly superior to the original models in all MRDx categories, although once again improvement of c-statistics was relatively small and majority of c-statistics of the enhanced models were within 95% confidence intervals of the original Elixhauser, except for acute myocardial infarction (Table 5).

**Table 5:** **Results of original Elixhauser models**^**1 **^**and enhanced Elixhauser**^**2 **^**models**

**MRDx**^**3**^	**c statistics**		**Hosmer-Lemeshow test**	
	**Elixhauser**^**4**^	**Enhanced**^**5**^	**Elixhauser**^**4**^	**Enhanced**^**5**^
	**(95%CI**^**6**^**)**	**(95%CI**^**6**^**)**	**chi-square (*P*)**	**chi-square (*P*)**
ICH^7^	0.736	0.748	7.3 (0.50)	7.8 (0.46)
	(0.723-0.749)	(0.736-0.760)		
Pneumonia	0.917	0.920	26.8 (<0.01)	26.8 (<0.01)
	(0.912-0.922)	(0.915-0.925)		
Ischemic infarct	0.787	0.805	14.6 (0.07)	17.2 (0.03)
	(0.767-0.807)	(0.786-0.824)		
AMI^8^	0.809	0.825	35.8 (<0.01)	35.4 (<0.01)
	(0.795-0.823)	(0.811-0.839)		
Non-alcoholic liver disease	0.798	0.811	24.1 (<0.01)	25.4 (<0.01)
	(0.782-0.814)	(0.796-0.826)		
Intracranial injury	0.778	0.790	12.3 (0.14)	13.5 (0.10)
	(0.759-0.797)	(0.771-0.809)		
CRF^9^	0.832	0.840	19.3 (0.01)	28.0 (<0.01)
	(0.816-0.848)	(0.825-0.855)		
COPD^10^	0.810	0.815	8.7 (0.37)	12.2 (0.14)
	(0.789-0.831)	(0.795-0.835)		
Alcoholic liver disease	0.777	0.788	8.9 (0.35)	3.2 (0.92)
	(0.753-0.801)	(0.764-0.812)		
Aspiration pneumonia	0.730	0.734	3.2 (0.92)	8.1 (0.42)
	(0.703-0.757)	(0.707-0.761)		
CHF^11^	0.699	0.707	3.5 (0.90)	4.7 (0.79)
	(0.672-0.726)	(0.680-0.734)		
Coronary atherosclerosis	0.881	0.889	22.7 (<0.01)	14.2 (0.08)
	(0.862-0.900)	(0.869-0.909)		

1 & 2 Multiple logistic regression models for predicting in-hospital mortalities composed of age + sex + status of health insurance + admission category (emergent or not) + operation (yes or no) + presence of each Elixhauser comorbidity (yes or no), before (Elixhauser models) and after (enhanced Elixhauser models) adding comorbidities inferred by drug prescription information, 3 Most responsive diagnoses, 4 Elixhauser models, 5 Enhanced Elixhauser models, 6 95% confidence interval calculated by bootstrapping, 7 Intracranial hemorrhage, 8 Acute myocardial infarction, 9 Chronic renal failure, 10 Chronic obstructive pulmonary disease, 11 Congestive heart failure.

## Discussion

In this study, we showed that the predictability of comorbidity-adjusted models can be improved by the addition of missed comorbidities inferred from patients’ medication data.

The Chronic Disease Score (CDS) has also attempted to use medication data to improve the predictive power of the comorbidity index. However, in the CDS, there is little consideration for drugs used to treat two or more diseases [26,27]. For example, epilepsy is considered as a comorbid condition in the CDS, yet many antiepileptic drugs are used to treat other conditions such as neurogenic pain and mood disorders. Consequently, inaccurate inference of comorbid conditions can occur, which may negatively affect predictability in the CDS system. In this study, we used drugs that are used exclusively to treat one disease category. Thus, in our study, we eliminated inaccurate inferences of comorbid conditions caused by drugs that are used to treat two or more conditions.

Other researchers have also tried to combine diagnosis-based scores (ex. Charlson index) and pharmacy-based scores (ex. CDS-1 or CDS-2). These studies showed that the predictabilities of combined models were generally better than those of models composed only of diagnosis-based scores [29,40,41]. Yet, in our opinion, these attempts at combined models had additional problems. First, these models created the potential for double counting and/or different counting of risk scores for cases with the same comorbidity. For example, if both patient A and patient B have rheumatoid arthritis (RA) and are prescribed gold salt to relieve RA, but the diagnosis of RA is missed only in the claim data of patient A, the risk scores of the two patients would be different even though they have the same comorbidity. In this example, the same pharmacy-based scores are assigned to both patient A and patient B because they take same medication, but the diagnosis-based score is assigned only to patient B. Second, by combining diagnosis-based scores and pharmacy-based scores, the number of independent variables (i.e., explanatory variables in a prediction model) inevitably increases. An increased number of independent variables may result in the instability of a prediction model, especially when the number of study cases is relatively small.

In our study, we used the medication data only to infer missed comorbidities, and inferred comorbidities were directly included in comorbidity-adjusted models without increasing the number of independent variables. Consequently, our study is free from the above two problems.

To find missed comorbidities, we included only those drugs that are used to treat a single disease entity. One potential concern was that this method would result in an uneven distribution of additional comorbidities inferred from the medication data. For example, the majority of patients with rheumatologic or connective tissue diseases have been prescribed immunosuppressants. Because such agents might be used to treat solid tumors or hematologic malignancies, we did not use immunosuppressants to infer missed comorbidities. As a result, the number of drug-inferred comorbidities of rheumatologic or connective tissue diseases was relatively small (Tables 2 and 4). Therefore, it would be reasonable to suspect that the uneven detection of missed comorbidities might decrease the fitness of the models. However, our study showed that fitness, as shown by the Hosmer-Lemeshow Goodness-of-Fit tests, was similar between the original and enhanced Charlson models as well as between the original and enhanced Elixhauser models (Tables 3 and 5). Thus, we could affirm that uneven detection of missed comorbidities did not constitute an unacceptable problem.

Another remarkable finding of our study is that using medication data to infer missed comorbidities improved the predictabilities of Elixhauser models more than those of Charlson models. We believe that these differences were related to the respective characteristics of the Charlson and Elixhauser models. In the Charlson index, the sum of the weight of comorbidities is used to adjust the severity of comorbidities without consideration to the interaction of MRDx and the specific comorbidity [17]. In the Elixhauser index, however, interaction between MRDx and each comorbidity is considered [18]. As a result, inferred comorbidities might increase the predictabilities of Elixhauser models more than those of Charlson models.

Another important consideration is that although we used the DRG screening method to differentiate between drug-inferred diagnoses that were POA and those that were non-POA, it is possible that POA conditions defined by DRG screening method could truly be non-POA conditions. However, in our study, the majority of drug-inferred comorbidities were chronic conditions that were not thought to develop after hospitalization (Tables 2 and 4). Thus, we thought that most of the drug-inferred comorbidities were developed before or at the time of hospitalization.

Our study has some limitation: first, we excluded cancer cases and cases diagnosed to have a wide range of clinical spectrums which occupied major portion of in-hospital mortalities, thus further studies are warranted to generalize the findings of our study; second, improvement of c-statistics in enhanced models was relatively small and most of c-statistics of enhanced models were within 95% confidence intervals of original models, thus to confirm the results of our study, additional studies should be followed.

## Conclusion

In conclusion, predictabilities of comorbidity-adjusted models might be improved by the use of medication data to infer missed comorbidities.

## Competing interests

The authors declare that they have no competing interests.

## Authors’ contributions

JHB: The main researcher, has analyzed the data and written the manuscript. SHH has processed the raw data. EJL has reviewed previous studies and related researches YK has designed framework of the study and supervised the entire study. All authors read and approved the final manuscript.

## Pre-publication history

The pre-publication history for this paper can be accessed here:

http://www.biomedcentral.com/1472-6947/13/128/prepub

## Supplementary Material

Additional file 1 Detailed information and model coefficients. Click here for file
