# Supplementary material for: The predictability of claim-data-based comorbidity-adjusted models could be improved by using medication data
Source: BMC Med Inform Decis Mak. 2013 Nov 20;13:128. doi: 10.1186/1472-6947-13-128 (PMC3842675; doi:10.1186/1472-6947-13-128)
Supplement: Additional file 1 — Detailed information and model coefficients. [file 1472-6947-13-128-S1.docx]

**Table S1. Charlson index with Deyo’s application**

| **Comorbid condition** | **Weight** | **Conditions required past medical data^1^** |  |
| --- | --- | --- | --- |
| Myocardial infarction | 1 | Acute myocardial infacrtion | |
| Congestive heart failure | 1 | All | |
| Peripheral vascular disease | 1 | Blood vessel replaced by prosthesis | |
| Cerebrovascular disease | 1 | Unless late effects of cerebrovascular disease | |
| Dementia | 1 | – | |
| Chronic pulmonary disease | 1 | – | |
| Connective tissue disease / rheumatic disease | 1 | – | |
| Peptic ulcer disease | 1 | All | |
| Mild liver disease | 1 | – | |
| Diabetes without complication | 1 | – | |
| Diabetes with complication | 2 | – | |
| Paraplegia & hemiplegia | 2 | – | |
| Renal disease | 2 | – | |
| Cancer | 3 | All | |
| Moderate or severe liver disease | 3 | – | |
| Metastatic carcinoma | 6 | All | |
| AIDS | 6 | – | |
| 1. Only if the condition(s) is (or are) documented during past admission at least last one or more year(s), is (or are) considered as comorbitiy (or comorbidities). | | |  |

**Table S2. Elixhauser comorbidity**

| **Diagnostic category** | **DRG^1^ screening^2^** |  |
| --- | --- | --- |
| Congestive heart failure | Cardiac | |
| Cardiac arrhythmias | Cardiac | |
| Valvular disease | Cardiac | |
| Pulmonary circulation disorders | Cardiac or COPD^3^ | |
| Peripheral vascular disorders | Peripheral vascular | |
| Hypertension, uncomplicated | Hypertension | |
| Hypertension, complicated | Hypertension or cardiac or renal | |
| Paralysis | Cerebrovascular | |
| Other neurological disorders | Nervous system | |
| Chronic pulmonary disease | COPD^3^ or asthma | |
| Diabetes, uncomplicated | Diabetes | |
| Diabetes, complicated | Diabetes | |
| Hypothyroidism | Thyroid or endocrine | |
| Renal failure | Kidney transplant or renal failure / dialysis | |
| Liver disease | Liver | |
| Peptic ulcer disease excluding bleeding | Gastrointestinal hemorrhage or ulcer | |
| AIDS | HIV | |
| Lymphoma | Leukemia / lymphoma | |
| Metastatic cancer | Cancer | |
| Solid tumor without metastasis | Cancer | |
| Rheumatoid arthritis / collagen vascular diseases | Connective tissue | |
| Coagulopathy | Coagulation | |
| Obesity | Obesity procedure or nutrition / metabolic | |
| Weight loss | Nutrition / metabolic | |
| Fluid and electrolyte disorders | Nutrition / metabolic | |
| Blood loss anemia | Anemia | |
| Deficiency anemia | Anemia | |
| Alcohol abuse | Alcohol or drug | |
| Drug abuse | Alcohol or drug | |
| Psychoses | Psychoses | |
| Depression | Depression | |
| 1. Diagnosis-related group, 2. By DRG screening, associated disease(s) which might be classified same disease category with the most responsible diagnosis (MRDx) and/or resulted from MRDx are considered as non-Present-On-Admission(s), 3. Chronic obstructive pulmonary disease. | |  |

**Table S3. Medications used to infer Charlson index comorbidities**

| **Comorbidities inferred by medication data** | **Drugs used to infer missed comorbidities** |  |
| --- | --- | --- |
| Congestive heart failure | Denopamine, digoxin, ubidecarenone | |
| Peripheral vascular disease | Cilostazol, sarpogrelate | |
| Cerebrovascular disease | Citicoline, ibudilast | |
| Dementia | Galantamine, memantine, rivastigmine | |
| Chronic pulmonary disease | Acepifylline, aminophylline, bambuterol, bamifylline, budesonide inhaler, budesonide / formoterol inhaler, ciclesonide inhaler, clenbuterol, doxofylline, fenoterol, fenoterol inhaler, fluticasone inhaler, fluticasone / salbutamol inhaler, formoterol, formoterol inhaler, hexaprenaline, ipratropium, ipratropium / salbutamol inhaler, montelukast, ozagrel, pranlukast, procaterol, procaterol inhaler, salbutamol, salbutamol inhaler, theophylline, tiotropium inhaler, zafirlukast | |
| Connective tissue disease / rheumatic disease | Auranofin, bucillamine, leflunomide | |
| Renal disease | Peritoneal dialysates | |
| AIDS | Abacavir, atazanavir, didanosine, efavirenz, indinavir, lamivudine (150mg), lopinavir / ritonavir, nelfinavir, nevirapine, ritonavir, stavudine, zidovudine | |
|  |  | |

**Table S4. Medications used to infer Elixhauer comorbidities**

| **Comorbidities inferred by medication data** | **Drugs used to infer missed comorbidities** |  |
| --- | --- | --- |
| Congestive heart failure | Denopamine, digoxin, ubidecarenone | |
| Cardiac arrhythmia | Propafenone, amiodarone, flecainide, mexiletine | |
| Peripheral vascular disorders | Cilostazol, sarpogrelate | |
| Other neurological disorders | Benztropine, biperiden, clonazepam, dihydroergocryptine, entacapone, ethosuximide, levetiracetam, levodopa / benserazide, levodopa/carbidopa, levodopa / carbidopa / entacapone, oxcarbazepine, pramipexole, procyclidine, selegiline, tetrabenazine, trihexyphenidyl, vigabatrin, zonisamide | |
| Chronic pulmonary disease | Acepifylline, aminophylline, bambuterol, bamifylline, budesonide inhaler, budesonide/formoterol inhaler, ciclesonide inhaler, clenbuterol, doxofylline, fenoterol, fenoterol inhaler, fluticasone inhaler, fluticasone/salbutamol inhaler, formoterol, formoterol inhaler, hexaprenaline, ipratropium, ipratropium/salbutamol inhaler, montelukast, ozagrel, pranlukast, procaterol, procaterol inhaler, salbutamol, salbutamol inhaler, theophylline, tiotropium inhaler, zafirlukast | |
| Hypothyroidism | Levothyroxine, liothyronine, levothyroxine / liothyronine | |
| Renal failure | Peritoneal dialysates | |
| Liver disease | Adefovir, clevudine, entecavir, lamivudine(100mg), malotilate | |
| AIDS | Abacavir, atazanavir, didanosine, efavirenz, indinavir, lamivudine (150mg), lopinavir / ritonavir, nelfinavir, nevirapine, ritonavir, stavudine, zidovudine | |
| Rheumatoid arthritis / collagen vascular diseases | Auranofin, bucillamine, leflunomide | |
| Weight loss | Megestrol | |
| Deficiency anemia | Ferric hydroxide-polymaltose complex, ferrous citrate, ferrous sulfate, iron acetyl-transferrin, iron protein succinylate, polysaccharide iron complex | |
| Psychoses | Amisulpride, bromperidol, haloperidol, nemonapride, olanzapine, quetiapine, risperidone, trifluoperazine, ziprasidone, zotepine | |
| Depression | Amoxapine, bupropion, dothiepin, mianserin, milnacipran, mirtazapine, quinupramine, tianeptin, trazodone | |
|  |  | |

**Table S5. Model coefficients of Charlson model for intracranial hemorrhage**

|  | β-coefficient | | p-value | Odds Ratio  (95% confidence interval) |  |
| --- | --- | --- | --- | --- | --- |
| Intercept | -4.0682 | |  |  | |
| Age | 0.0152 | | <.0001 | 1.015 (1.012 - 1.019) | |
| Male | 0.1032 | | 0.0571 | 1.109 (0.997 - 1.233) | |
| Medical Aids | 0.4167 | | <.0001 | 1.517 (1.268 - 1.815) | |
| Emergency admission | 1.4004 | | <.0001 | 4.045 (4.345 - 4.791) | |
| Operation | 0.1177 | | 0.0312 | 1.125 (1.011 -1.252) | |
| Charlson index score | -0.0524 | | 0.0046 | 1.054 (1.016 - 1.093) | |
|  | |  | | | |

**Table S6. Model coefficients of Charlson model for pneumonia**

|  | β-coefficient | | p-value | Odds Ratio  (95% confidence interval) | | |  |
| --- | --- | --- | --- | --- | --- | --- | --- |
| Intercept | -8.3513 | | <.0001 |  |  |  | |
| Age | 0.0524 | | <.0001 | 1.054 (1.050 - 1.057) | | | |
| Male | 0.3405 | | <.0001 | 1.406 (1.246 - 1.586) | | | |
| Medical Aids | 0.2561 | | 0.0029 | 1.292 (1.091 - 1.529) | | | |
| Emergency admission | 0.7844 | | <.0001 | 2.191 (1.915 - 2.507) | | | |
| Operation | 0.8015 | | <.0001 | 2.229 (1.797 - 2.765) | | | |
| Charlson index score | 0.1007 | | <.0001 | 1.106 (1.080 - 1.132) | | | |
|  | |  | | | | | |

**Table S7. Model coefficients of Charlson model for ischemic infarct**

|  | β-coefficient | | p-value | Odds Ratio  (95% confidence interval) | | |  |
| --- | --- | --- | --- | --- | --- | --- | --- |
| Intercept | -7.2495 | | <.0001 |  |  |  | |
| Age | 0.0394 | | <.0001 | 1.040 (1.033 - 1.047) | | | |
| Male | -0.1761 | | 0.0134 | 0.839 (0.729 - 0.964) | | | |
| Medical Aids | 0.1917 | | 0.0935 | 1.211 (0.968 - 1.515) | | | |
| Emergency admission | 1.0213 | | <.0001 | 2.777 (2.264 - 3.406) | | | |
| Operation | 1.6517 | | <.0001 | 5.216 (4.348 - 6.257) | | | |
| Charlson index score | 0.0691 | | 0.0004 | 1.072 (1.032 - 1.113) | | | |
|  | |  | | | | | |

**Table S8. Model coefficients of Charlson model for acute myocardial infarction**

|  | β-coefficient | | p-value | Odds Ratio  (95% confidence interval) | | |  |
| --- | --- | --- | --- | --- | --- | --- | --- |
| Intercept | -7.2495 | | <.0001 |  |  |  | |
| Age | 0.0394 | | <.0001 | 1.040 (1.033 - 1.047) | | | |
| Male | -0.1761 | | 0.0134 | 0.839 (0.729 - 0.964) | | | |
| Medical Aids | 0.1917 | | 0.0935 | 1.211 (0.968 - 1.515) | | | |
| Emergency admission | 1.0213 | | <.0001 | 2.777 (2.264 - 3.406) | | | |
| Operation | 1.6517 | | <.0001 | 5.216 (4.348 - 6.257) | | | |
| Charlson index score | 0.0691 | | 0.0004 | 1.072 (1.032 - 1.113) | | | |
|  | |  | | | | | |

**Table S9. Model coefficients of Charlson model for non-alcoholic liver disease**

|  | β-coefficient | | p-value | Odds Ratio  (95% confidence interval) | | |  |
| --- | --- | --- | --- | --- | --- | --- | --- |
| Intercept | -7.1776 | | <.0001 |  |  |  | |
| Age | 0.0173 | | <.0001 | 1.017 (1.012 - 1.023) | | | |
| Male | 0.1753 | | 0.033 | 1.192 (1.014 - 1.400) | | | |
| Medical Aids | 0.4849 | | <.0001 | 1.624 (1.344 - 1.963) | | | |
| Emergency admission | 0.8871 | | <.0001 | 2.428 (2.050 - 2.876) | | | |
| Operation | -0.0431 | | 0.6983 | 0.958 (0.770 - 1.191) | | | |
| Charlson index score | 0.2082 | | <.0001 | 1.231 (1.197 - 1.266) | | | |
|  | |  | | | | | |

**Table S10. Model coefficients of Charlson model for intracranial injury**

|  | β-coefficient | | p-value | Odds Ratio  (95% confidence interval) | | |  |
| --- | --- | --- | --- | --- | --- | --- | --- |
| Intercept | -7.0633 | | <.0001 |  |  |  | |
| Age | 0.0223 | | <.0001 | 1.023 (1.018 - 1.027) | | | |
| Male | 0.4495 | | <.0001 | 1.568 (1.288 - 1.908) | | | |
| Medical Aids | 0.3194 | | 0.0150 | 1.376 (1.064 - 1.780) | | | |
| Emergency admission | 1.3723 | | <.0001 | 3.944 (2.917 - 5.334) | | | |
| Operation | 0.6491 | | <.0001 | 1.914 (1.614 - 2.269) | | | |
| Charlson index score | 0.1579 | | <.0001 | 1.171 (1.102 - 1.245) | | | |
|  | |  | | | | | |

**Table S11. Model coefficients of Charlson model for chronic renal failure**

|  | β-coefficient | | p-value | Odds Ratio  (95% confidence interval) | | |  |
| --- | --- | --- | --- | --- | --- | --- | --- |
| Intercept | -7.5476 | | <.0001 |  |  |  | |
| Age | 0.0414 | | <.0001 | 1.042 (1.035 - 1.050) | | | |
| Male | 0.1337 | | 0.1324 | 1.143 (0.960 - 1.360) | | | |
| Medical Aids | 0.2032 | | 0.0537 | 1.225 (0.997 - 1.506) | | | |
| Emergency admission | 0.6463 | | <.0001 | 1.908 (1.600 - 2.276) | | | |
| Operation | -0.9575 | | <.0001 | 0.384 (0.296 - 0.497) | | | |
| Charlson index score | 0.1965 | | <.0001 | 1.217 (1.167 - 1.269) | | | |
|  | |  | | | | | |

**Table S12. Model coefficients of Charlson model for chronic obstructive pulmonary disease**

|  | β-coefficient | | p-value | Odds Ratio  (95% confidence interval) | | |  |
| --- | --- | --- | --- | --- | --- | --- | --- |
| Intercept | -7.8591 | | <.0001 |  |  |  | |
| Age | 0.0459 | | <.0001 | 1.047 (1.037 - 1.057) | | | |
| Male | 0.3472 | | 0.0026 | 1.415 (1.129 - 1.774) | | | |
| Medical Aids | 0.0838 | | 0.5444 | 1.087 (0.829 - 1.426) | | | |
| Emergency admission | 0.7828 | | <.0001 | 2.188 (1.745 - 2.742) | | | |
| Operation | 0.5868 | | 0.0025 | 1.798 (1.228 - 2.632) | | | |
| Charlson index score | 0.1414 | | <.0001 | 1.152 (1.092 - 1.215) | | | |
|  | |  | | | | | |

**Table S13. Model coefficients of Charlson model for alcoholic liver disease**

|  | β-coefficient | | p-value | Odds Ratio  (95% confidence interval) | | |  |
| --- | --- | --- | --- | --- | --- | --- | --- |
| Intercept | -4.9784 | | <.0001 |  |  |  | |
| Age | 0.0141 | | 0.0046 | 1.014 (1.004 - 1.024) | | | |
| Male | -0.1891 | | 0.2250 | 0.828 (0.610 - 1.123) | | | |
| Medical Aids | 0.0505 | | 0.6882 | 1.052 (0.822 - 1.346) | | | |
| Emergency admission | 1.0074 | | <.0001 | 2.739 (2.140 - 3.504) | | | |
| Operation | 0.0426 | | 0.7468 | 1.044 (0.806 - 1.352) | | | |
| Charlson index score | 0.1750 | | <.0001 | 1.191 (1.144 - 1.241) | | | |
|  | |  | | | | | |

**Table S14. Model coefficients of Charlson model for aspiration pneumonia**

|  | β-coefficient | | p-value | Odds Ratio  (95% confidence interval) | | |  |
| --- | --- | --- | --- | --- | --- | --- | --- |
| Intercept | -5.3510 | | <.0001 |  |  |  | |
| Age | 0.0318 | | <.0001 | 1.032 (1.024 - 1.040) | | | |
| Male | 0.0378 | | 0.7528 | 1.039 (0.821 - 1.314) | | | |
| Medical Aids | 0.2845 | | 0.0779 | 1.329 (0.969 - 1.824) | | | |
| Emergency admission | 0.2131 | | 0.1161 | 1.237 (0.949 - 1.614) | | | |
| Operation | 0.1961 | | 0.3549 | 1.345 (0.718 - 2.518) | | | |
| Charlson index score | 0.0281 | | 0.2778 | 1.028 (0.978 - 1.082) | | | |
|  | |  | | | | | |

**Table S15. Model coefficients of Charlson model for congestive heart failure**

|  | β-coefficient | | p-value | Odds Ratio  (95% confidence interval) | | |  |
| --- | --- | --- | --- | --- | --- | --- | --- |
| Intercept | -4.7071 | | <.0001 |  |  |  | |
| Age | 0.0166 | | 0.0004 | 1.017 (1.008 - 1.026) | | | |
| Male | 0.3987 | | 0.0004 | 1.490 (1.210 - 1.835) | | | |
| Medical Aids | -0.1861 | | 0.2425 | 0.830 (0.608 - 1.134) | | | |
| Emergency admission | 0.5456 | | <.0001 | 1.726 (1.357 - 2.194) | | | |
| Operation | -0.5426 | | 0.00439 | 0.581 (0.402 - 0.840) | | | |
| Charlson index score | 0.1098 | | <.0001 | 1.116 (1.059 - 1.177) | | | |
|  | |  | | | | | |

**Table S16. Model coefficients of Charlson model for coronary atherosclerosis**

|  | β-coefficient | | p-value | Odds Ratio  (95% confidence interval) | | |  |
| --- | --- | --- | --- | --- | --- | --- | --- |
| Intercept | -9.0945 | | <.0001 |  |  |  | |
| Age | 0.0548 | | <.0001 | 1.056 (1.044 - 1.069) | | | |
| Male | 0.0434 | | 0.7229 | 1.044 (0.821 - 1.328) | | | |
| Medical Aids | -0.0161 | | 0.9302 | 0.984 (0.686 - 1.411) | | | |
| Emergency admission | 1.0270 | | <.0001 | 2.793 (2.158 - 3.614) | | | |
| Operation | -1.1910 | | <.0001 | 0.304 (0.235 - 0.393) | | | |
| Charlson index score | 0.3073 | | <.0001 | 1.360 (1.294 - 1.428) | | | |
|  | |  | | | | | |

**Table S17. Model coefficients of enhanced Charlson model for intracramoal hemorrhage**

|  | β-coefficient | | p-value | Odds Ratio  (95% confidence interval) | | |  |
| --- | --- | --- | --- | --- | --- | --- | --- |
| Intercept | -4.0779 | | <.0001 |  |  |  | |
| Age | 0.0151 | | <.0001 | 1.015 (1.011 - 1.019) | | | |
| Male | 0.0999 | | 0.0656 | 1.105 (0.994 - 1.229) | | | |
| Medical Aids | 0.4125 | | <.0001 | 1.511 (1.263 - 1.807) | | | |
| Emergency admission | 1.4033 | | <.0001 | 4.069 (3.445 - 4.805) | | | |
| Operation | 0.1193 | | 0.0291 | 1.127 (1.012 - 1.254) | | | |
| Charlson index score | -0.0355 | | 0.0456 | 0.965 (0.932 - 0.999) | | | |
|  | |  | | | | | |

**Table S18. Model coefficients of enhanced Charlson model for pneumonia**

|  | β-coefficient | | p-value | Odds Ratio  (95% confidence interval) | | |  |
| --- | --- | --- | --- | --- | --- | --- | --- |
| Intercept | -7.3645 | | <.0001 |  |  |  | |
| Age | 0.0521 | | <.0001 | 1.053 (1.050 - 1.057) | | | |
| Male | 0.3319 | | <.0001 | 1.394 (1.235 - 1.572) | | | |
| Medical Aids | 0.2485 | | 0.0039 | 1.282 (1.083 - 1.518) | | | |
| Emergency admission | 0.7817 | | <.0001 | 2.185 (1.909 - 2.501) | | | |
| Operation | 0.8054 | | <.0001 | 2.238 (1.803 - 2.777) | | | |
| Charlson index score | 0.1174 | | <.0001 | 1.125 (1.099 - 1.151) | | | |
|  | |  | | | | | |

**Table S19. Model coefficients of enhanced Charlson model for ischemic infarct**

|  | β-coefficient | | p-value | Odds Ratio  (95% confidence interval) | | |  |
| --- | --- | --- | --- | --- | --- | --- | --- |
| Intercept | -7.2557 | | <.0001 |  |  |  | |
| Age | 0.0393 | | <.0001 | 1.040 (1.033 - 1.047) | | | |
| Male | -0.1759 | | 0.0135 | 0.839 (0.729 - 0.964) | | | |
| Medical Aids | 0.193 | | 0.0912 | 1.213 (0.970 - 1.517) | | | |
| Emergency admission | 1.0213 | | <.0001 | 2.777 (2.264 - 3.406) | | | |
| Operation | 1.6507 | | <.0001 | 5.211 (4.344 - 6.251) | | | |
| Charlson index score | 0.0681 | | 0.0003 | 1.070 (1.032 - 1.111) | | | |
|  | |  | | | | | |

**Table S20. Model coefficients of enhanced Charlson model for acute myocardial infarct**

|  | β-coefficient | | p-value | Odds Ratio  (95% confidence interval) | | |  |
| --- | --- | --- | --- | --- | --- | --- | --- |
| Intercept | -6.7907 | | <.0001 |  |  |  | |
| Age | 0.0577 | | <.0001 | 1.059 (1.052 - 1.067) | | | |
| Male | 0.0519 | | 0.5084 | 1.053 (0.903 - 1.228) | | | |
| Medical Aids | 0.0935 | | 0.4076 | 1.098 (0.880 - 1.370) | | | |
| Emergency admission | 0.4735 | | <.0001 | 1.606 (1.309 - 1.969) | | | |
| Operation | -0.99 | | <.0001 | 0.372 (0.320 - 0.431) | | | |
| Charlson index score | 0.1916 | | <.0001 | 1.211 (1.165- 1.259) | | | |
|  | |  | | | | | |

**Table S21. Model coefficients of enhanced Charlson model for non-alcoholic liver disease**

|  | β-coefficient | | p-value | Odds Ratio  (95% confidence interval) | | |  |
| --- | --- | --- | --- | --- | --- | --- | --- |
| Intercept | -5.2598 | | <.0001 |  |  |  | |
| Age | 0.0167 | | <.0001 | 1.017 (1.011 - 1.022) | | | |
| Male | 0.1689 | | 0.0405 | 1.184 (1.007 - 1.392) | | | |
| Medical Aids | 0.4746 | | <.0001 | 1.607 (1.329 - 1.943) | | | |
| Emergency admission | 0.8841 | | <.0001 | 2.421 (2.043 - 2.869) | | | |
| Operation | -0.0707 | | 0.5257 | 0.932 (0.749 - 1.159) | | | |
| Charlson index score | 0.2248 | | <.0001 | 1.252 (1.218 - 1.2870 | | | |
|  | |  | | | | | |

**Table S22. Model coefficients of enhanced Charlson model for intracranial injury**

|  | β-coefficient | | p-value | Odds Ratio  (95% confidence interval) | | |  |
| --- | --- | --- | --- | --- | --- | --- | --- |
| Intercept | 5.7884 | | <.0001 |  |  |  | |
| Age | -0.0221 | | <.0001 | 0.978 (0.974 - 0.983) | | | |
| Male | -0.4528 | | <.0001 | 0.636 (0.523 - 0.774) | | | |
| Medical Aids | -0.3195 | | 0.0149 | 0.727 (0.562 - 0.940) | | | |
| Emergency admission | -1.3764 | | <.0001 | 0.252 (0.187 - 0.341) | | | |
| Operation | -0.649 | | <.0001 | 0.523 (0.441 - 0.620) | | | |
| Charlson index score | -0.1531 | | <.0001 | 0.858 (0.809 - 0.911) | | | |
|  | |  | | | | | |

**Table S23. Model coefficients of enhanced Charlson model for chronic renal failure**

|  | β-coefficient | | p-value | Odds Ratio  (95% confidence interval) | | |  |
| --- | --- | --- | --- | --- | --- | --- | --- |
| Intercept | -6.7544 | | <.0001 |  |  |  | |
| Age | 0.0409 | | <.0001 | 1.042 (1.034 - 1.049) | | | |
| Male | 0.1313 | | 0.1395 | 1.140 (0.958 - 1.357) | | | |
| Medical Aids | 0.1955 | | 0.0635 | 1.216 (0.989 - 1.495) | | | |
| Emergency admission | 0.6387 | | <.0001 | 1.894 (1.588 - 2.259) | | | |
| Operation | -0.9617 | | <.0001 | 0.382 (0.295 - 0.495) | | | |
| Charlson index score | 0.2045 | | <.0001 | 1.227 (1.178 - 1.278) | | | |
|  | |  | | | | | |

**Table S24. Model coefficients of enhanced Charlson model for chronic obstructive pulmonary disease**

|  | β-coefficient | | p-value | Odds Ratio  (95% confidence interval) | | |  |
| --- | --- | --- | --- | --- | --- | --- | --- |
| Intercept | -7.5283 | | <.0001 |  |  |  | |
| Age | 0.0451 | | <.0001 | 1.046 (1.036 - 1.056) | | | |
| Male | 0.3438 | | 0.0029 | 1.410 (1.125 - 1.769) | | | |
| Medical Aids | 0.0783 | | 0.5716 | 1.081 (0.825 - 1.418) | | | |
| Emergency admission | 0.7879 | | <.0001 | 2.199 (1.754 - 2.757) | | | |
| Operation | 0.5919 | | 0.0024 | 1.807 (1.234 - 2.648) | | | |
| Charlson index score | 0.1707 | | <.0001 | 1.186 (1.128 - 1.247) | | | |
|  | |  | | | | | |

**Table S25. Model coefficients of enhanced Charlson model for alcoholic liver disease**

|  | β-coefficient | | p-value | Odds Ratio  (95% confidence interval) | | |  |
| --- | --- | --- | --- | --- | --- | --- | --- |
| Intercept | -4.8180 | | <.0001 |  |  |  | |
| Age | 0.0134 | | 0.0074 | 1.013 (1.004 - 1.023) | | | |
| Male | -0.1925 | | 0.2176 | 0.825 (0.607 - 1.120) | | | |
| Medical Aids | 0.0525 | | 0.6768 | 1.054 (0.823 - 1.349) | | | |
| Emergency admission | 1.0023 | | <.0001 | 2.725 (2.129 - 3.487) | | | |
| Operation | 0.0146 | | 0.9117 | 1.015 (0.784 - 1.314) | | | |
| Charlson index score | 0.1977 | | <.0001 | 1.219 (1.171 - 1.268) | | | |
|  | |  | | | | | |

**Table S26. Model coefficients of enhanced Charlson model for aspiration pneumonia**

|  | β-coefficient | | p-value | Odds Ratio  (95% confidence interval) | | |  |
| --- | --- | --- | --- | --- | --- | --- | --- |
| Intercept | -4.2317 | | <.0001 |  |  |  | |
| Age | 0.0317 | | <.0001 | 1.032 (1.024 - 1.040) | | | |
| Male | 0.0363 | | 0.7624 | 1.037 (0.819 - 1.313) | | | |
| Medical Aids | 0.2836 | | 0.079 | 1.328 (0.968 - 1.822) | | | |
| Emergency admission | 0.2161 | | 0.1108 | 1.241 (0.952 - 1.619) | | | |
| Operation | 0.2957 | | 0.3556 | 1.344 (0.718 - 2.517) | | | |
| Charlson index score | 0.0359 | | 0.1612 | 1.037 (0.986 - 1.090) | | | |
|  | |  | | | | | |

**Table S27. Model coefficients of enhanced Charlson model for congestive heart failure**

|  | β-coefficient | | p-value | Odds Ratio  (95% confidence interval) | | |  |
| --- | --- | --- | --- | --- | --- | --- | --- |
| Intercept | -4.8580 | | <.0001 |  |  |  | |
| Age | 0.0164 | | 0.0004 | 1.017 (1.007 - 1.026) | | | |
| Male | 0.3868 | | 0.0003 | 1.472 (1.195 - 1.814) | | | |
| Medical Aids | -0.1964 | | 0.2178 | 0.822 (0.601 - 1.123) | | | |
| Emergency admission | 0.5404 | | <.0001 | 1.717 (1.350 - 2.183) | | | |
| Operation | -0.5435 | | 0.0038 | 0.581 (0.402 - 0.839) | | | |
| Charlson index score | 0.1348 | | <.0001 | 1.144 (1.088 - 1.203) | | | |
|  | |  | | | | | |

**Table S28. Model coefficients of enhanced Charlson model for coronary atherosclerosis**

|  | β-coefficient | | p-value | Odds Ratio  (95% confidence interval) | | |  |
| --- | --- | --- | --- | --- | --- | --- | --- |
| Intercept | -9.1723 | | <.0001 |  |  |  | |
| Age | 0.0536 | | <.0001 | 1.055 (1.043 - 1.068) | | | |
| Male | 0.0198 | | 0.8723 | 1.020 (0.802 - 1.298) | | | |
| Medical Aids | -0.0486 | | 0.7926 | 0.953 (0.663 - 1.369) | | | |
| Emergency admission | 1.0304 | | <.0001 | 2.802 (2.166 - 3.625) | | | |
| Operation | -1.2047 | | <.0001 | 0.300 (0.232 - 0.387) | | | |
| Charlson index score | 0.3361 | | <.0001 | 1.400 (1.337 - 1.466) | | | |
|  | |  | | | | | |

**Table S29. Model coefficients of Elixhauser model for intracranial hemorrhage**

|  | | β-coefficient | p-value | Odds Ratio  (95% confidence interval) | | |  |
| --- | --- | --- | --- | --- | --- | --- | --- |
| Intercept | | -4.0108 | <.0001 |  |  |  | |
| Age | | 0.0173 | <.0001 | 1.017 (1.014 - 1.021) | | | |
| Medical Aids | | 0.334 | 0.0004 | 1.397 (1.159 - 1.682) | | | |
| Emergency admission | | 1.382 | <.0001 | 3.983 (3.365 - 4.714) | | | |
| Congestive heart failure | | 0.3558 | 0.0066 | 1.427 (1.104 - 1.845) | | | |
| Cardiac arrhythmias | | 0.2059 | 0.0483 | 1.229 (1.002 - 1.507) | | | |
| Hypertension, uncomplicated | | -0.6361 | <.0001 | 0.529 (0.473 - 0.592) | | | |
| Chronic pulmonary disease | | -0.6102 | <.0001 | 0.543 (0.445 - 0.663) | | | |
| Diabetes, uncomplicated | | 0.4684 | <.0001 | 1.597 (1.419 - 1.799) | | | |
| Renal failure | | 1.0221 | <.0001 | 2.779 (2.080 - 3.713) | | | |
| Peptic ulcer disease excluding bleeding | | -0.5969 | <.0001 | 0.551 (0.451 - 0.671) | | | |
| Lymphoma | | 2.6713 | 0.0082 | 14.458 (1.995 - 104.778) | | | |
| Metastatic cancer | | 1.6238 | <.0001 | 5.073 (2.531 - 10.165) | | | |
| Coagulopathy | | 1.0096 | <.0001 | 2.745 (2.270 - 3.319) | | | |
| Weight loss | | 0.2269 | 0.0192 | 1.255 (1.038 - 1.517) | | | |
| Fluid and electrolyte disorders | | 0.4343 | <.0001 | 1.544 (1.299 - 1.835) | | | |
| Psychoses | | -2.2086 | 0.0021 | 0.110 (0.027 - 0.448) | | | |
| Depression | | -2.2609 | <.0001 | 0.104 (0.046 - 0.235) | | | |
|  |  | | | | | | |

**Table S30. Model coefficients of Elixhauser model for pneumonia**

|  | | β-coefficient | p-value | Odds Ratio  (95% confidence interval) | | |  |
| --- | --- | --- | --- | --- | --- | --- | --- |
| Intercept | | -7.3501 | <.0001 |  |  |  | |
| Age | | 0.0505 | <.0001 | 1.052 (1.048 - 1.056) | | | |
| Sex | | 0.2683 | <.0001 | 1.308 (1.152 - 1.485) | | | |
| Medical Aids | | 0.2302 | 0.0106 | 1.259 (1.055 - 1.502) | | | |
| Emergency admission | | 0.6713 | <.0001 | 1.957 (1.701 - 2.251) | | | |
| Operation | | 0.7751 | <.0001 | 2.171 (1.726 - 2.730) | | | |
| Congestive heart failure | | 0.3908 | <.0001 | 1.478 (1.217 - 1.796) | | | |
| Cardiac arrhythmias | | 0.6951 | <.0001 | 2.004 (1.694 - 2.370) | | | |
| Pulmonary circulation disorders | | 0.5794 | <.0001 | 1.785 (1.363 - 2.337) | | | |
| Hypertension, uncomplicated | | -0.3638 | <.0001 | 0.695 (0.610 - 0.792) | | | |
| Other neurological disorders | | 0.6454 | <.0001 | 1.907 (1.566 - 2.322) | | | |
| Diabetes, uncomplicated | | 0.3736 | <.0001 | 1.453 (1.260 - 1.676) | | | |
| Diabetes, complicated | | 0.2436 | 0.0064 | 1.276 (1.071 - 1.520) | | | |
| Renal failure | | 0.4422 | 0.0011 | 1.556 (1.192 - 2.031) | | | |
| Peptic ulcer disease excluding bleeding | | -0.4581 | <.0001 | 0.632 (0.520 - 0.770) | | | |
| Metastatic cancer | | 0.4676 | 0.0141 | 1.596 (1.099 - 2.318) | | | |
| Solid tumor without metastasis | | -0.2662 | 0.0113 | 0.766 (0.624 - 0.942) | | | |
| Coagulopathy | | 1.5838 | <.0001 | 4.874 (4.023 - 5.904) | | | |
| Weight loss | | 0.7474 | <.0001 | 2.112 (1.782 - 2.502) | | | |
| Fluid and electrolyte disorders | | 0.7941 | <.0001 | 2.213 (1.903 - 2.573) | | | |
| Alcohol abuse | | 0.8117 | <.0001 | 2.252 (1.604 - 3.162) | | | |
| Depression | | -0.4273 | 0.0156 | 0.652 (0.461 - 0.922) | | | |
|  |  | | | | | | |

**Table S31. Model coefficients of Elixhauser model for ischemic infarct**

|  | | β-coefficient | p-value | Odds Ratio  (95% confidence interval) | | |  |
| --- | --- | --- | --- | --- | --- | --- | --- |
| Intercept | | -6.7317 | <.0001 |  |  |  | |
| Age | | 0.035 | <.0001 | 1.036 (1.029 - 1.042) | | | |
| Sex | | -0.1689 | 0.0206 | 0.845 (0.732 - 0.974) | | | |
| Emergency admission | | 0.9633 | <.0001 | 2.620 (2.130 - 3.224) | | | |
| Operation | | 1.5288 | <.0001 | 4.613 (3.814 - 5.580) | | | |
| Congestive heart failure | | 0.8491 | <.0001 | 2.338 (1.855 - 2.946) | | | |
| Cardiac arrhythmias | | 0.5307 | <.0001 | 1.700 (1.448 - 1.997) | | | |
| Hypertension, uncomplicated | | -0.4716 | <.0001 | 0.624 (0.541 - 0.719) | | | |
| Chronic pulmonary disease | | 0.5186 | <.0001 | 1.680 (1.367 - 2.063) | | | |
| Diabetes, complicated | | -0.4068 | 0.0001 | 0.666 (0.542 - 0.818) | | | |
| Renal failure | | 0.7565 | 0.0001 | 2.131 (1.443 - 3.146) | | | |
| Liver disease | | 0.3526 | 0.0013 | 1.423 (1.147 - 1.764) | | | |
| Peptic ulcer disease excluding bleeding | | -0.4561 | 0.0001 | 0.634 (0.502 - 0.801) | | | |
| Metastatic cancer | | 1.3844 | <.0001 | 3.992 (2.208 - 7.218) | | | |
| Rheumatoid arthritis / collagen vascular diseases | | -0.7587 | 0.0141 | 0.468 (0.255 - 0.858) | | | |
| Coagulopathy | | 1.0473 | <.0001 | 2.850 (2.153 - 3.773) | | | |
| Weight loss | | 0.6213 | <.0001 | 1.861 (1.381 - 2.508) | | | |
| Fluid and electrolyte disorders | | 0.9738 | <.0001 | 2.648 (2.118 - 3.310) | | | |
| Deficiency anemia | | -0.3321 | 0.0183 | 0.717 (0.544 - 0.945) | | | |
| Psychoses | | -1.3703 | 0.0031 | 0.254 (0.102 - 0.630) | | | |
| Depression | | -0.9044 | <.0001 | 0.405 (0.284 - 0.577) | | | |
|  |  | | | | | | |

**Table S32. Model coefficients of Elixhauser model for acute myocardial infarction**

|  | | β-coefficient | p-value | Odds Ratio  (95% confidence interval) | | |  |
| --- | --- | --- | --- | --- | --- | --- | --- |
| Intercept | | -6.3823 | <.0001 |  |  |  | |
| Age | | 0.0566 | <.0001 | 1.058 (1.051 - 1.066) | | | |
| Emergency admission | | 0.4627 | <.0001 | 1.588 (1.286 - 1.961) | | | |
| Operation | | -0.8598 | <.0001 | 0.423 (0.363 - 0.494) | | | |
| Peripheral vascular disorders | | -0.3881 | 0.0328 | 0.678 (0.475 - 0.969) | | | |
| Hypertension, uncomplicated | | -0.6204 | <.0001 | 0.538 (0.462 - 0.626) | | | |
| Other neurological disorders | | 1.3601 | <.0001 | 3.896 (2.928 - 5.185) | | | |
| Chronic pulmonary disease | | 0.2556 | 0.0101 | 1.291 (1.063 - 1.569) | | | |
| Diabetes, uncomplicated | | 0.2067 | 0.0086 | 1.230 (1.054 - 1.435) | | | |
| Renal failure | | 0.6783 | <.0001 | 1.970 (1.508 - 2.575) | | | |
| Peptic ulcer disease excluding bleeding | | -0.5067 | 0.0002 | 0.602 (0.460 - 0.789) | | | |
| Coagulopathy | | 1.0854 | <.0001 | 2.961 (2.193 - 3.997) | | | |
| Weight loss | | 0.8101 | <.0001 | 2.248 (1.575 - 3.208) | | | |
| Fluid and electrolyte disorders | | 0.7281 | <.0001 | 2.071 (1.694 - 2.532) | | | |
| Blood loss anemia | | 2.5899 | 0.001 | 13.328 (2.846 - 62.423) | | | |
|  |  | | | | | | |

**Table S33. Model coefficients of Elixhauser model for non-alcoholic liver disease**

|  | | β-coefficient | p-value | Odds Ratio  (95% confidence interval) | | |  |
| --- | --- | --- | --- | --- | --- | --- | --- |
| Intercept | | -5.1605 | <.0001 |  |  |  | |
| Age | | 0.0203 | <.0001 | 1.021 (1.015 - 1.026) | | | |
| Medical Aids | | 0.4169 | <.0001 | 1.517 (1.244 - 1.851) | | | |
| Emergency admission | | 0.7749 | <.0001 | 2.170 (1.820 - 2.587) | | | |
| Congestive heart failure | | 1.1814 | <.0001 | 3.259 (2.257 - 4.706) | | | |
| Pulmonary circulation disorders | | 1.3115 | 0.0088 | 3.712 (1.390 - 9.908) | | | |
| Hypertension, uncomplicated | | -0.5337 | <.0001 | 0.586 (0.467 - 0.736) | | | |
| Paralysis | | 0.8893 | 0.0353 | 2.433 (1.063 - 5.570) | | | |
| Other neurological disorders | | 0.4131 | 0.0105 | 1.511 (1.101 - 2.074) | | | |
| Chronic pulmonary disease | | 0.5387 | <.0001 | 1.714 (1.322 - 2.221) | | | |
| Diabetes, uncomplicated | | 0.3494 | <.0001 | 1.418 (1.191 - 1.689) | | | |
| Renal failure | | 0.6671 | 0.0012 | 1.949 (1.302 - 2.916) | | | |
| Peptic ulcer disease excluding bleeding | | -0.7545 | <.0001 | 0.470 (0.365 - 0.605) | | | |
| Lymphoma | | 1.4544 | 0.0164 | 4.282 (1.305 - 14.048) | | | |
| Metastatic cancer | | 0.9445 | 0.0004 | 2.571 (1.525 - 4.335) | | | |
| Solid tumor without metastasis | | -0.4556 | 0.001 | 0.634 (0.483 - 0.832) | | | |
| Coagulopathy | | 1.337 | <.0001 | 3.808 (3.168 - 4.576) | | | |
| Weight loss | | 0.3922 | 0.0024 | 1.480 (1.149 - 1.906) | | | |
| Fluid and electrolyte disorders | | 1.1058 | <.0001 | 3.022 (2.487 - 3.671) | | | |
| Blood loss anemia | | -0.2587 | 0.0355 | 0.772 (0.607 - 0.983) | | | |
| Alcohol abuse | | 0.6221 | <.0001 | 1.863 (1.443 - 2.404) | | | |
| Psychoses | | -1.9238 | 0.0176 | 0.146 (0.030 - 0.715) | | | |
|  |  | | | | | | |

**Table S34. Model coefficients of Elixhauser model for intracranial injury**

|  | | β-coefficient | p-value | Odds Ratio  (95% confidence interval) | | |  |
| --- | --- | --- | --- | --- | --- | --- | --- |
| Intercept | | 5.7394 | <.0001 |  |  |  | |
| Age | | -0.0233 | <.0001 | 0.977 (0.972 - 0.982) | | | |
| Sex | | -0.4385 | <.0001 | 0.645 (0.526 - 0.790) | | | |
| Medical Aids | | -0.3032 | 0.0267 | 0.738 (0.565 - 0.966) | | | |
| Emergency admission | | -1.2257 | <.0001 | 0.294 (0.216 - 0.399) | | | |
| Operation | | -0.5991 | <.0001 | 0.549 (0.460 - 0.656) | | | |
| Congestive heart failure | | -1.0082 | <.0001 | 0.365 (0.240 - 0.555) | | | |
| Cardiac arrhythmias | | -0.4111 | 0.0207 | 0.663 (0.468 - 0.939) | | | |
| Pulmonary circulation disorders | | -1.7358 | 0.0049 | 0.176 (0.053 - 0.591) | | | |
| Hypertension, uncomplicated | | 0.5098 | <.0001 | 1.665 (1.349 - 2.054) | | | |
| Paralysis | | 0.9599 | <.0001 | 2.611 (1.623 - 4.203) | | | |
| Chronic pulmonary disease | | 0.3824 | 0.0363 | 1.466 (1.025 - 2.097) | | | |
| Diabetes, uncomplicated | | -0.7115 | <.0001 | 0.491 (0.402 - 0.600) | | | |
| Diabetes, complicated | | -0.6933 | 0.0001 | 0.500 (0.350 - 0.715) | | | |
| Renal failure | | -0.8198 | 0.0036 | 0.441 (0.254 - 0.765) | | | |
| Peptic ulcer disease excluding bleeding | | 0.8217 | <.0001 | 2.274 (1.584 - 3.266) | | | |
| Coagulopathy | | -1.1805 | <.0001 | 0.307 (0.233 - 0.405) | | | |
| Fluid and electrolyte disorders | | -1.0273 | <.0001 | 0.358 (0.268 - 0.478) | | | |
| Depression | | 1.8184 | 0.0024 | 6.162 (1.901 - 19.970) | | | |
|  |  | | | | | | |

**Table S35. Model coefficients of Elixhauser model for chronic renal failure**

|  | | β-coefficient | p-value | Odds Ratio  (95% confidence interval) | | |  |
| --- | --- | --- | --- | --- | --- | --- | --- |
| Intercept | | -6.2462 | <.0001 |  |  |  | |
| Age | | 0.0378 | <.0001 | 1.038 (1.031 - 1.046) | | | |
| Emergency admission | | 0.6149 | <.0001 | 1.850 (1.540 - 2.221) | | | |
| Operation | | -0.9387 | <.0001 | 0.391 (0.299 - 0.511) | | | |
| Congestive heart failure | | 0.5898 | <.0001 | 1.804 (1.400 - 2.323) | | | |
| Cardiac arrhythmias | | 0.5049 | 0.0002 | 1.657 (1.265 - 2.170) | | | |
| Pulmonary circulation disorders | | 0.8384 | 0.0304 | 2.313 (1.083 - 4.940) | | | |
| Hypertension, uncomplicated | | -0.3935 | <.0001 | 0.675 (0.558 - 0.815) | | | |
| Hypertension, complicated | | -0.5441 | 0.0017 | 0.580 (0.413 - 0.815) | | | |
| Paralysis | | 1.089 | <.0001 | 2.971 (1.912 - 4.618) | | | |
| Other neurological disorders | | 1.3783 | <.0001 | 3.968 (3.116 - 5.052) | | | |
| Chronic pulmonary disease | | 0.5243 | <.0001 | 1.689 (1.347 - 2.118) | | | |
| Liver disease | | 0.4644 | 0.0002 | 1.591 (1.248 - 2.028) | | | |
| Peptic ulcer disease excluding bleeding | | -0.3631 | 0.0188 | 0.696 (0.514 - 0.941) | | | |
| Rheumatoid arthritis / collagen vascular diseases | | -1.1038 | 0.0247 | 0.332 (0.127 - 0.869) | | | |
| Coagulopathy | | 1.993 | <.0001 | 7.338 (5.612 - 9.594) | | | |
| Weight loss | | 0.7851 | <.0001 | 2.193 (1.600 - 3.006) | | | |
|  |  | | | | | | |

**Table S36. Model coefficients of Elixhauser model for chronic obasturctive pulmonary disease**

|  | | β-coefficient | p-value | Odds Ratio  (95% confidence interval) | | |  |
| --- | --- | --- | --- | --- | --- | --- | --- |
| Intercept | | -7.2449 | <.0001 |  |  |  | |
| Age | | 0.0386 | <.0001 | 1.039 (1.029 - 1.049) | | | |
| Sex | | 0.3686 | 0.002 | 1.446 (1.144 - 1.828) | | | |
| Emergency admission | | 0.6248 | <.0001 | 1.868 (1.478 - 2.361) | | | |
| Operation | | 0.5203 | 0.0119 | 1.683 (1.122 - 2.524) | | | |
| Congestive heart failure | | 0.744 | <.0001 | 2.104 (1.537 - 2.880) | | | |
| Cardiac arrhythmias | | 0.781 | <.0001 | 2.184 (1.659 - 2.875) | | | |
| Hypertension, uncomplicated | | -0.2439 | 0.0364 | 0.784 (0.624 - 0.985) | | | |
| Hypertension, complicated | | -0.9947 | 0.0046 | 0.370 (0.186 - 0.736) | | | |
| Other neurological disorders | | 0.8681 | <.0001 | 2.382 (1.566 - 3.624) | | | |
| Diabetes, uncomplicated | | 0.6508 | <.0001 | 1.917 (1.507 - 2.439) | | | |
| Diabetes, complicated | | 0.4122 | 0.0182 | 1.510 (1.073 - 2.126) | | | |
| Renal failure | | 0.7855 | 0.0039 | 2.193 (1.286 - 3.741) | | | |
| Peptic ulcer disease excluding bleeding | | -0.4862 | 0.0089 | 0.615 (0.427 - 0.885) | | | |
| Solid tumor without metastasis | | -0.4817 | 0.0315 | 0.618 (0.398 - 0.958) | | | |
| Coagulopathy | | 1.4788 | <.0001 | 4.388 (2.905 - 6.629) | | | |
| Weight loss | | 0.9938 | <.0001 | 2.702 (2.013 - 3.626) | | | |
| Fluid and electrolyte disorders | | 0.9998 | <.0001 | 2.718 (2.070 - 3.568) | | | |
|  |  | | | | | | |

**Table S37. Model coefficients of Elixhauser model for alcoholic liver disease**

|  | | β-coefficient | p-value | Odds Ratio  (95% confidence interval) | | |  |
| --- | --- | --- | --- | --- | --- | --- | --- |
| Intercept | | -4.711 | <.0001 |  |  |  | |
| Age | | 0.0172 | 0.0006 | 1.017 (1.007 - 1.027) | | | |
| Emergency admission | | 0.8819 | <.0001 | 2.415 (1.877 - 3.109) | | | |
| Congestive heart failure | | 1.0948 | <.0001 | 2.989 (1.791 - 4.988) | | | |
| Hypertension, uncomplicated | | -0.6368 | 0.0003 | 0.529 (0.376 - 0.744) | | | |
| Other neurological disorders | | 0.4741 | 0.0073 | 1.607 (1.136 - 2.272) | | | |
| Chronic pulmonary disease | | 0.5153 | 0.0054 | 1.674 (1.165 - 2.407) | | | |
| Peptic ulcer disease excluding bleeding | | -0.8888 | <.0001 | 0.411 (0.289 - 0.585) | | | |
| Metastatic cancer | | 0.9897 | 0.0121 | 2.690 (1.241 - 5.830) | | | |
| Solid tumor without metastasis | | -0.5212 | 0.0024 | 0.594 (0.424 - 0.831) | | | |
| Coagulopathy | | 1.3681 | <.0001 | 3.928 (3.106 - 4.968) | | | |
| Weight loss | | 0.3481 | 0.0368 | 1.416 (1.022 - 1.964) | | | |
| Fluid and electrolyte disorders | | 0.7726 | <.0001 | 2.165 (1.675 - 2.799) | | | |
| Alcohol abuse | | -0.6912 | 0.0003 | 0.501 (0.344 - 0.730) | | | |
| Depression | | -1.2636 | 0.0141 | 0.283 (0.103 - 0.775) | | | |
|  |  | | | | | | |

**Table S38. Model coefficients of Elixhauser model for aspiration pneumonia**

|  | | β-coefficient | p-value | Odds Ratio  (95% confidence interval) | | |  |
| --- | --- | --- | --- | --- | --- | --- | --- |
| Intercept | | -4.0206 | <.0001 |  |  |  | |
| Age | | 0.0329 | <.0001 | 1.033 (1.026 - 1.041) | | | |
| Congestive heart failure | | 0.9572 | <.0001 | 2.604 (1.783 - 3.805) | | | |
| Hypertension, uncomplicated | | -0.3573 | 0.0027 | 0.700 (0.554 - 0.883) | | | |
| Paralysis | | -0.9972 | <.0001 | 0.369 (0.227 - 0.599) | | | |
| Renal failure | | 0.7487 | 0.0019 | 2.114 (1.318 - 3.391) | | | |
| Peptic ulcer disease excluding bleeding | | -0.3692 | 0.0446 | 0.691 (0.482 - 0.991) | | | |
| Metastatic cancer | | 1.2617 | 0.0005 | 3.532 (1.729 - 7.214) | | | |
| Coagulopathy | | 1.0706 | <.0001 | 2.917 (2.006 - 4.242) | | | |
| Fluid and electrolyte disorders | | 0.562 | <.0001 | 1.754 (1.326 - 2.320) | | | |
| Psychoses | | -1.4449 | 0.002 | 0.236 (0.094 - 0.590) | | | |
|  |  | | | | | | |

**Table S39. Model coefficients of Elixhauser model for congestive heart failure**

|  | | β-coefficient | p-value | Odds Ratio  (95% confidence interval) | | |  |
| --- | --- | --- | --- | --- | --- | --- | --- |
| Intercept | | -4.8218 | <.0001 |  |  |  | |
| Age | | 0.0194 | <.0001 | 1.020 (1.010 - 1.029) | | | |
| Sex | | 0.4209 | <.0001 | 1.523 (1.233 - 1.881) | | | |
| Emergency admission | | 0.5225 | <.0001 | 1.686 (1.322 - 2.151) | | | |
| Operation | | -0.472 | 0.0126 | 0.624 (0.430 - 0.904) | | | |
| Hypertension, uncomplicated | | -0.5704 | <.0001 | 0.565 (0.458 - 0.697) | | | |
| Other neurological disorders | | 0.7731 | 0.0008 | 2.167 (1.378 - 3.406) | | | |
| Liver disease | | 0.3176 | 0.0372 | 1.374 (1.019 - 1.852) | | | |
| Peptic ulcer disease excluding bleeding | | -0.5027 | 0.0128 | 0.605 (0.407 - 0.899) | | | |
| Metastatic cancer | | 0.8171 | 0.0442 | 2.264 (1.022 - 5.018) | | | |
| Coagulopathy | | 0.8554 | <.0001 | 2.352 (1.553 - 3.563) | | | |
| Obesity | | 0.9772 | <.0001 | 2.657 (1.770 - 3.989) | | | |
| Fluid and electrolyte disorders | | 0.8258 | <.0001 | 2.284 (1.779 - 2.932) | | | |
|  |  | | | | | | |

**Table S40. Model coefficients of Elixhauser model for coronary athersclerosis**

|  | | β-coefficient | p-value | Odds Ratio  (95% confidence interval) | | |  |
| --- | --- | --- | --- | --- | --- | --- | --- |
| Intercept | | -8.554 | <.0001 |  |  |  | |
| Age | | 0.0502 | <.0001 | 1.051 (1.039 - 1.064) | | | |
| Emergency admission | | 0.8468 | <.0001 | 2.332 (1.793 - 3.034) | | | |
| Operation | | -1.1905 | <.0001 | 0.304 (0.234 - 0.396) | | | |
| Hypertension, uncomplicated | | -0.6598 | <.0001 | 0.517 (0.403 - 0.663) | | | |
| Other neurological disorders | | 0.8527 | 0.0009 | 2.346 (1.416 - 3.887) | | | |
| Chronic pulmonary disease | | 0.5328 | 0.0009 | 1.704 (1.243 - 2.334) | | | |
| Diabetes, uncomplicated | | 0.3563 | 0.0072 | 1.428 (1.101 - 1.852) | | | |
| Renal failure | | 1.3942 | <.0001 | 4.032 (2.868 - 5.667) | | | |
| Liver disease | | 0.4194 | 0.0184 | 1.521 (1.073 - 2.156) | | | |
| Peptic ulcer disease excluding bleeding | | -0.5604 | 0.0087 | 0.571 (0.376 - 0.868) | | | |
| Metastatic cancer | | 1.0271 | 0.0339 | 2.793 (1.081 - 7.217) | | | |
| Coagulopathy | | 1.8252 | <.0001 | 6.204 (4.221 - 9.118) | | | |
| Weight loss | | 1.5471 | <.0001 | 4.698 (2.923 - 7.552) | | | |
| Fluid and electrolyte disorders | | 1.1326 | <.0001 | 3.104 (2.262 - 4.259) | | | |
| Deficiency anemia | | 0.388 | 0.0332 | 1.474 (1.031 - 2.106) | | | |
|  |  | | | | | | |

**Table S41. Model coefficients of enhanced Elixhauser model for intracranial hemorrhage**

|  | | β-coefficient | p-value | Odds Ratio  (95% confidence interval) | | |  |
| --- | --- | --- | --- | --- | --- | --- | --- |
| Intercept | | -3.9812 | <.0001 |  |  |  | |
| Age | | 0.0176 | <.0001 | 1.018 (1.014 - 1.022) | | | |
| Medical Aids | | 0.3231 | 0.0007 | 1.381 (1.145 - 1.666) | | | |
| Emergency admission | | 1.3876 | <.0001 | 4.005 (3.382 - 4.743) | | | |
| Congestive heart failure | | 0.3354 | 0.0043 | 1.398 (1.111 - 1.760) | | | |
| Hypertension, uncomplicated | | -0.6386 | <.0001 | 0.528 (0.472 - 0.591) | | | |
| Chronic pulmonary disease | | -0.5435 | <.0001 | 0.581 (0.486 - 0.694) | | | |
| Diabetes, uncomplicated | | 0.4743 | <.0001 | 1.607 (1.426 - 1.811) | | | |
| Renal failure | | 1.1649 | <.0001 | 3.206 (2.472 - 4.157) | | | |
| Peptic ulcer disease excluding bleeding | | -0.5818 | <.0001 | 0.559 (0.458 - 0.682) | | | |
| Lymphoma | | 2.6425 | 0.0093 | 14.048 (1.920 - 102.81) | | | |
| Metastatic cancer | | 1.6551 | <.0001 | 5.233 (2.592 - 10.567) | | | |
| Coagulopathy | | 1.0077 | <.0001 | 2.739 (2.262 - 3.317) | | | |
| Weight loss | | 0.2468 | 0.011 | 1.280 (1.058 - 1.548) | | | |
| Fluid and electrolyte disorders | | 0.4032 | <.0001 | 1.497 (1.257 - 1.782) | | | |
| Psychoses | | -2.1441 | <.0001 | 0.117 (0.058 - 0.238) | | | |
| Depression | | -2.3357 | <.0001 | 0.097 (0.048 - 0.196) | | | |
|  |  | | | | | | |

**Table S42. Model coefficients of enhanced Elixhauser model for pneumonia**

|  | | β-coefficient | p-value | Odds Ratio  (95% confidence interval) | | |  |
| --- | --- | --- | --- | --- | --- | --- | --- |
| Intercept | | -7.4335 | <.0001 |  |  |  | |
| Age | | 0.0503 | <.0001 | 1.052 (1.048 - 1.055) | | | |
| Sex | | 0.242 | 0.0002 | 1.274 (1.121 - 1.447) | | | |
| Medical Aids | | 0.2069 | 0.022 | 1.230 (1.030 - 1.468) | | | |
| Emergency admission | | 0.6785 | <.0001 | 1.971 (1.713 - 2.268) | | | |
| Operation | | 0.7717 | <.0001 | 2.163 (1.719 - 2.723) | | | |
| Congestive heart failure | | 0.2993 | 0.0009 | 1.349 (1.131 - 1.609) | | | |
| Cardiac arrhythmias | | 0.6454 | <.0001 | 1.907 (1.606 - 2.264) | | | |
| Pulmonary circulation disorders | | 0.5369 | 0.0001 | 1.711 (1.302 - 2.248) | | | |
| Hypertension, uncomplicated | | -0.3987 | <.0001 | 0.671 (0.588 - 0.766) | | | |
| Other neurological disorders | | 0.5815 | <.0001 | 1.789 (1.478 - 2.165) | | | |
| Chronic pulmonary disease | | 0.1564 | 0.0156 | 1.169 (1.030 - 1.327) | | | |
| Diabetes, uncomplicated | | 0.3736 | <.0001 | 1.453 (1.259 - 1.676) | | | |
| Diabetes, complicated | | 0.1998 | 0.0261 | 1.221 (1.024 - 1.456) | | | |
| Renal failure | | 0.9424 | <.0001 | 2.566 (2.061 - 3.196) | | | |
| Peptic ulcer disease excluding bleeding | | -0.4355 | <.0001 | 0.647 (0.532 - 0.787) | | | |
| Metastatic cancer | | 0.4327 | 0.0229 | 1.541 (1.062 - 2.238) | | | |
| Solid tumor without metastasis | | -0.2777 | 0.0084 | 0.758 (0.616 - 0.931) | | | |
| Coagulopathy | | 1.5609 | <.0001 | 4.763 (3.926 - 5.778) | | | |
| Weight loss | | 0.7412 | <.0001 | 2.099 (1.779 - 2.476) | | | |
| Fluid and electrolyte disorders | | 0.756 | <.0001 | 2.130 (1.830 - 2.479) | | | |
| Alcohol abuse | | 0.8362 | <.0001 | 2.307 (1.643 - 3.242) | | | |
| Depression | | -0.5058 | 0.0023 | 0.603 (0.436 - 0.834) | | | |
|  |  | | | | | | |

**Table S43. Model coefficients of enhanced Elixhauser model for ischemic infarct**

|  | | β-coefficient | p-value | Odds Ratio  (95% confidence interval) | | |  |
| --- | --- | --- | --- | --- | --- | --- | --- |
| Intercept | | -6.617 | <.0001 |  |  |  | |
| Age | | 0.0344 | <.0001 | 1.035 (1.028 - 1.042) | | | |
| Sex | | -0.1604 | 0.0293 | 0.852 (0.737 - 0.984) | | | |
| Emergency admission | | 0.9701 | <.0001 | 2.638 (2.142 - 3.250) | | | |
| Operation | | 1.5175 | <.0001 | 4.561 (3.762 - 5.530) | | | |
| Congestive heart failure | | 0.7257 | <.0001 | 2.066 (1.691 - 2.525) | | | |
| Cardiac arrhythmias | | 0.3952 | <.0001 | 1.485 (1.254 - 1.757) | | | |
| Peripheral vascular disorders | | -0.8929 | <.0001 | 0.409 (0.317 - 0.529) | | | |
| Hypertension, uncomplicated | | -0.4416 | <.0001 | 0.643 (0.557 - 0.742) | | | |
| Chronic pulmonary disease | | 0.6632 | <.0001 | 1.941 (1.606 - 2.345) | | | |
| Diabetes, complicated | | -0.3973 | 0.0002 | 0.672 (0.546 - 0.827) | | | |
| Renal failure | | 1.2259 | <.0001 | 3.407 (2.442 - 4.754) | | | |
| Liver disease | | 0.3188 | 0.004 | 1.375 (1.107 - 1.709) | | | |
| Peptic ulcer disease excluding bleeding | | -0.4364 | 0.0003 | 0.646 (0.511 - 0.818) | | | |
| Metastatic cancer | | 1.2951 | <.0001 | 3.652 (2.010 - 6.635) | | | |
| Rheumatoid arthritis / collagen vascular diseases | | -0.7814 | 0.0135 | 0.458 (0.246 - 0.851) | | | |
| Coagulopathy | | 1.0271 | <.0001 | 2.793 (2.108 - 3.701) | | | |
| Weight loss | | 0.6532 | <.0001 | 1.922 (1.432 - 2.579) | | | |
| Fluid and electrolyte disorders | | 0.9257 | <.0001 | 2.524 (2.013 - 3.164) | | | |
| Deficiency anemia | | -0.3375 | 0.0117 | 0.714 (0.549 - 0.928) | | | |
| Psychoses | | -1.0439 | <.0001 | 0.352 (0.210 - 0.591) | | | |
| Depression | | -0.9322 | <.0001 | 0.394 (0.283 - 0.547) | | | |
|  |  | | | | | | |

**Table S44. Model coefficients of enhanced Elixhauser model for acute myocardial infarction**

|  | | β-coefficient | p-value | Odds Ratio  (95% confidence interval) | | |  |
| --- | --- | --- | --- | --- | --- | --- | --- |
| Intercept | | -6.3311 | <.0001 |  |  |  | |
| Age | | 0.0546 | <.0001 | 1.056 | 1.049 | 1.063 | |
| Sex | | 0.4795 | <.0001 | 1.615 | 1.304 | 2.001 | |
| Emergency admission | | -0.7319 | <.0001 | 0.481 | 0.41 | 0.564 | |
| Peripheral vascular disorders | | -0.5744 | <.0001 | 0.563 | 0.458 | 0.692 | |
| Hypertension, uncomplicated | | -0.6154 | <.0001 | 0.54 | 0.464 | 0.63 | |
| Other neurological disorders | | 1.288 | <.0001 | 3.625 | 2.736 | 4.804 | |
| Chronic pulmonary disease | | 0.3923 | <.0001 | 1.48 | 1.24 | 1.767 | |
| Diabetes, uncomplicated | | 0.2027 | 0.0108 | 1.225 | 1.048 | 1.431 | |
| Renal failure | | 1.205 | <.0001 | 3.337 | 2.67 | 4.171 | |
| Peptic ulcer disease excluding bleeding | | -0.4965 | 0.0003 | 0.609 | 0.464 | 0.799 | |
| Coagulopathy | | 0.9883 | <.0001 | 2.687 | 1.984 | 3.637 | |
| Weight loss | | 0.7928 | <.0001 | 2.21 | 1.564 | 3.122 | |
| Fluid and electrolyte disorders | | 0.6321 | <.0001 | 1.882 | 1.538 | 2.303 | |
| Blood loss anemia | | 2.4634 | 0.0015 | 11.745 | 2.555 | 53.982 | |
|  |  | | | | | | |

**Table S45. Model coefficients of enhanced Elixhauser model for non-alcoholic liver disease**

|  | | β-coefficient | p-value | Odds Ratio  (95% confidence interval) | | |  |
| --- | --- | --- | --- | --- | --- | --- | --- |
| Intercept | | -5.1476 | <.0001 |  |  |  | |
| Age | | 0.0191 | <.0001 | 1.019 (1.014 - 1.025) | | | |
| Medical Aids | | 0.4054 | <.0001 | 1.500 (1.227 - 1.833) | | | |
| Emergency admission | | 0.776 | <.0001 | 2.173 (1.821 - 2.593) | | | |
| Congestive heart failure | | 1.1544 | <.0001 | 3.172 (2.286 - 4.401) | | | |
| Pulmonary circulation disorders | | 1.1381 | 0.0239 | 3.121 (1.162 - 8.379) | | | |
| Hypertension, uncomplicated | | -0.6086 | <.0001 | 0.544 (0.432 - 0.685) | | | |
| Paralysis | | 0.9099 | 0.0318 | 2.484 (1.082 - 5.702) | | | |
| Other neurological disorders | | 0.3652 | 0.0199 | 1.441 (1.059 - 1.960) | | | |
| Chronic pulmonary disease | | 0.7403 | <.0001 | 2.097 (1.677 - 2.621) | | | |
| Diabetes, uncomplicated | | 0.3336 | 0.0002 | 1.396 (1.171 - 1.665) | | | |
| Renal failure | | 1.2264 | <.0001 | 3.409 (2.463 - 4.718) | | | |
| Peptic ulcer disease excluding bleeding | | -0.7817 | <.0001 | 0.458 (0.354 - 0.591) | | | |
| Lymphoma | | 1.2909 | 0.0371 | 3.636 (1.080 - 12.239) | | | |
| Metastatic cancer | | 0.9277 | 0.0005 | 2.529 (1.495 - 4.278) | | | |
| Solid tumor without metastasis | | -0.4275 | 0.002 | 0.652 (0.497 - 0.855) | | | |
| Coagulopathy | | 1.3148 | <.0001 | 3.724 (3.094 - 4.483) | | | |
| Weight loss | | 0.4803 | 0.0001 | 1.616 (1.267 - 2.062) | | | |
| Fluid and electrolyte disorders | | 1.0793 | <.0001 | 2.943 (2.418 - 3.582) | | | |
| Deficiency anemia | | -0.3023 | 0.0113 | 0.739 (0.585 - 0.934) | | | |
| Alcohol abuse | | 0.609 | <.0001 | 1.839 (1.422 - 2.378) | | | |
| Psychoses | | -1.181 | 0.0021 | 0.307 (0.145 - 0.652) | | | |
|  |  | | | | | | |

**Table S46. Model coefficients of enhanced Elixhauser model for intracranial injury**

|  | | β-coefficient | p-value | Odds Ratio  (95% confidence interval) | | |  |
| --- | --- | --- | --- | --- | --- | --- | --- |
| Intercept | | 5.7248 | <.0001 |  |  |  | |
| Age | | -0.0235 | <.0001 | 0.977 (0.972 - 0.982) | | | |
| Sex | | -0.4462 | <.0001 | 0.640 (0.522 - 0.784) | | | |
| Medical Aids | | -0.3171 | 0.0209 | 0.728 (0.556 - 0.953) | | | |
| Emergency admission | | -1.2213 | <.0001 | 0.295 (0.217 - 0.401) | | | |
| Operation | | -0.6385 | <.0001 | 0.528 (0.442 - 0.631) | | | |
| Congestive heart failure | | -0.6951 | 0.0004 | 0.499 (0.340 - 0.733) | | | |
| Pulmonary circulation disorders | | -1.6111 | 0.0121 | 0.200 (0.057 - 0.703) | | | |
| Hypertension, uncomplicated | | 0.5334 | <.0001 | 1.705 (1.381 - 2.104) | | | |
| Paralysis | | 0.9519 | <.0001 | 2.591 (1.608 - 4.173) | | | |
| Diabetes, uncomplicated | | -0.7284 | <.0001 | 0.483 (0.394 - 0.591) | | | |
| Diabetes, complicated | | -0.7284 | <.0001 | 0.483 (0.337 - 0.691) | | | |
| Renal failure | | -0.9354 | 0.0002 | 0.392 (0.240 - 0.640) | | | |
| Peptic ulcer disease excluding bleeding | | 0.7843 | <.0001 | 2.191 (1.530 - 3.137) | | | |
| Coagulopathy | | -1.2233 | <.0001 | 0.294 (0.223 - 0.388) | | | |
| Fluid and electrolyte disorders | | -1.0265 | <.0001 | 0.358 (0.268 - 0.479) | | | |
| Psychoses | | 2.4888 | <.0001 | 12.047 (3.813 - 38.068) | | | |
| Depression | | 1.6682 | 0.0012 | 5.303 (1.935 - 14.533) | | | |
|  |  | | | | | | |

**Table S47. Model coefficients of enhanced Elixhauser model for chronic renal failure**

|  | | β-coefficient | p-value | Odds Ratio  (95% confidence interval) | | |  |
| --- | --- | --- | --- | --- | --- | --- | --- |
| Intercept | | -6.0369 | <.0001 |  |  |  | |
| Age | | 0.0352 | <.0001 | 1.036 (1.029 - 1.043) | | | |
| Emergency admission | | 0.5916 | <.0001 | 1.807 (1.503 - 2.172) | | | |
| Operation | | -0.897 | <.0001 | 0.408 (0.312 - 0.534) | | | |
| Congestive heart failure | | 0.3889 | 0.0013 | 1.475 (1.164 - 1.869) | | | |
| Cardiac arrhythmias | | 0.4672 | 0.0005 | 1.596 (1.224 - 2.079) | | | |
| Hypertension, uncomplicated | | -0.3899 | <.0001 | 0.677 (0.559 - 0.820) | | | |
| Hypertension, complicated | | -0.4797 | 0.0055 | 0.619 (0.441 - 0.869) | | | |
| Paralysis | | 1.133 | <.0001 | 3.105 (1.995 - 4.832) | | | |
| Other neurological disorders | | 1.141 | <.0001 | 3.130 (2.487 - 3.939) | | | |
| Chronic pulmonary disease | | 0.8476 | <.0001 | 2.334 (1.906 - 2.858) | | | |
| Liver disease | | 0.4446 | 0.0003 | 1.560 (1.223 - 1.990) | | | |
| Peptic ulcer disease excluding bleeding | | -0.3244 | 0.0359 | 0.723 (0.534 - 0.979) | | | |
| Rheumatoid arthritis / collagen vascular diseases | | -1.0298 | 0.0293 | 0.357 (0.141 - 0.901) | | | |
| Coagulopathy | | 2.0076 | <.0001 | 7.445 (5.688 - 9.746) | | | |
| Weight loss | | 0.8111 | <.0001 | 2.250 (1.668 - 3.036) | | | |
| Fluid and electrolyte disorders | | 0.2271 | 0.0256 | 1.255 (1.028 - 1.532) | | | |
| Deficiency anemia | | -0.4411 | <.0001 | 0.643 (0.534 - 0.775) | | | |
|  |  | | | | | | |

**Table S48. Model coefficients of enhanced Elixhauser model for chronic obstructive pulmonary disease**

|  | | β-coefficient | p-value | Odds Ratio  (95% confidence interval) | | |  |
| --- | --- | --- | --- | --- | --- | --- | --- |
| Intercept | | -7.2092 | <.0001 |  |  |  | |
| Age | | 0.0374 | <.0001 | 1.038 (1.028 - 1.048) | | | |
| Sex | | 0.3621 | 0.0026 | 1.436 (1.135 - 1.818) | | | |
| Emergency admission | | 0.6661 | <.0001 | 1.947 (1.539 - 2.462) | | | |
| Operation | | 0.5047 | 0.0152 | 1.656 (1.102 - 2.489) | | | |
| Congestive heart failure | | 0.6735 | <.0001 | 1.961 (1.488 - 2.584) | | | |
| Cardiac arrhythmias | | 0.6705 | <.0001 | 1.955 (1.472 - 2.597) | | | |
| Hypertension, uncomplicated | | -0.3136 | 0.008 | 0.731 (0.580 - 0.922) | | | |
| Hypertension, complicated | | -0.9855 | 0.0038 | 0.373 (0.191 - 0.728) | | | |
| Other neurological disorders | | 0.6597 | 0.0012 | 1.934 (1.299 - 2.880) | | | |
| Diabetes, uncomplicated | | 0.6327 | <.0001 | 1.883 (1.478 - 2.398) | | | |
| Diabetes, complicated | | 0.4 | 0.0223 | 1.492 (1.059 - 2.102) | | | |
| Renal failure | | 1.3321 | <.0001 | 3.789 (2.440 - 5.883) | | | |
| Peptic ulcer disease excluding bleeding | | -0.5267 | 0.0053 | 0.591 (0.408 - 0.855) | | | |
| Solid tumor without metastasis | | -0.49 | 0.0292 | 0.613 (0.394 - 0.952) | | | |
| Coagulopathy | | 1.4877 | <.0001 | 4.427 (2.931 - 6.685) | | | |
| Weight loss | | 0.9367 | <.0001 | 2.551 (1.902 - 3.422) | | | |
| Fluid and electrolyte disorders | | 0.933 | <.0001 | 2.542 (1.930 - 3.349) | | | |
| Psychoses | | 0.5388 | 0.0189 | 1.714 (1.093 - 2.687) | | | |
|  |  | | | | | | |

**Table S49. Model coefficients of enhanced Elixhauser model for alcoholic liver disease**

|  | | β-coefficient | p-value | Odds Ratio  (95% confidence interval) | | |  |
| --- | --- | --- | --- | --- | --- | --- | --- |
| Intercept | | -4.6118 | <.0001 |  |  |  | |
| Age | | 0.0134 | 0.0089 | 1.013 (1.003 - 1.024) | | | |
| Emergency admission | | 0.8755 | <.0001 | 2.400 (1.860 - 3.098) | | | |
| Congestive heart failure | | 0.9565 | 0.0001 | 2.603 (1.589 - 4.262) | | | |
| Hypertension, uncomplicated | | -0.7349 | <.0001 | 0.480 (0.339 - 0.679) | | | |
| Other neurological disorders | | 0.3916 | 0.0236 | 1.479 (1.054 - 2.077) | | | |
| Chronic pulmonary disease | | 0.8047 | <.0001 | 2.236 (1.636 - 3.057) | | | |
| Diabetes, uncomplicated | | 0.2317 | 0.049 | 1.261 (1.001 - 1.588) | | | |
| Renal failure | | 1.4224 | <.0001 | 4.147 (2.675 - 6.4290 | | | |
| Peptic ulcer disease excluding bleeding | | -0.8791 | <.0001 | 0.415 (0.291 - 0.593) | | | |
| Metastatic cancer | | 0.8281 | 0.0416 | 2.289 (1.032 - 5.077) | | | |
| Solid tumor without metastasis | | -0.5683 | 0.0011 | 0.566 (0.402 - 0.798) | | | |
| Coagulopathy | | 1.3128 | <.0001 | 3.717 (2.930 - 4.714) | | | |
| Weight loss | | 0.3352 | 0.0445 | 1.398 (1.008 - 1.939) | | | |
| Fluid and electrolyte disorders | | 0.6653 | <.0001 | 1.945 (1.497 - 2.528) | | | |
| Alcohol abuse | | -0.613 | 0.0015 | 0.542 (0.371 - 0.790) | | | |
| Depression | | -1.2643 | 0.0031 | 0.282 (0.122 - 0.653) | | | |
|  |  | | | | | | |

**Table S50. Model coefficients of enhanced Elixhauser model for aspiration pneumonia**

|  | | β-coefficient | p-value | Odds Ratio  (95% confidence interval) | | |  |
| --- | --- | --- | --- | --- | --- | --- | --- |
| Intercept | | -3.9872 | <.0001 |  |  |  | |
| Age | | 0.033 | <.0001 | 1.034 (1.026 - 1.042) | | | |
| Congestive heart failure | | 0.717 | <.0001 | 2.048 (1.481 - 2.833) | | | |
| Hypertension, uncomplicated | | -0.3706 | 0.002 | 0.690 (0.546 - 0.873) | | | |
| Paralysis | | -0.9571 | 0.0001 | 0.384 (0.237 - 0.623) | | | |
| Renal failure | | 0.9381 | <.0001 | 2.555 (1.661 - 3.931) | | | |
| Peptic ulcer disease excluding bleeding | | -0.3815 | 0.0389 | 0.683 (0.475 - 0.981) | | | |
| Metastatic cancer | | 1.2838 | 0.0005 | 3.619 (1.758 - 7.416) | | | |
| Coagulopathy | | 0.9987 | <.0001 | 2.715 (1.860 - 3.962) | | | |
| Fluid and electrolyte disorders | | 0.5499 | 0.0001 | 1.733 (1.307 - 2.297) | | | |
| Psychoses | | -0.9381 | 0.0006 | 0.391 (0.228 - 0.671) | | | |
| Depression | | -0.5578 | 0.0399 | 0.572 (0.336 - 0.975) | | | |
|  |  | | | | | | |

**Table S51. Model coefficients of enhanced Elixhauser model for congestive heart failure**

|  | | β-coefficient | p-value | Odds Ratio  (95% confidence interval) | | |  |
| --- | --- | --- | --- | --- | --- | --- | --- |
| Intercept | | -4.7941 | <.0001 |  |  |  | |
| Age | | 0.0173 | 0.0002 | 1.017 (1.008 - 1.027) | | | |
| Sex | | 0.3902 | 0.0003 | 1.477 (1.195 - 1.827) | | | |
| Emergency admission | | 0.4952 | <.0001 | 1.641 (1.286 - 2.094) | | | |
| Operation | | -0.484 | 0.0107 | 0.616 (0.425 - 0.8940 | | | |
| Hypertension, uncomplicated | | -0.5802 | <.0001 | 0.560 (0.454 - 0.6910 | | | |
| Other neurological disorders | | 0.6241 | 0.0031 | 1.867 (1.235 - 2.822) | | | |
| Chronic pulmonary disease | | 0.3937 | 0.0003 | 1.482 (1.197 - 1.837) | | | |
| Renal failure | | 0.3494 | 0.0228 | 1.418 (1.050 - 1.916) | | | |
| Liver disease | | 0.3266 | 0.0318 | 1.386 (1.029 - 1.868) | | | |
| Peptic ulcer disease excluding bleeding | | -0.5409 | 0.0075 | 0.582 (0.392 - 0.865) | | | |
| Coagulopathy | | 0.8702 | <.0001 | 2.387 (1.574 - 3.621) | | | |
| Weight loss | | 0.9462 | <.0001 | 2.576 (1.732 - 3.832) | | | |
| Fluid and electrolyte disorders | | 0.7594 | <.0001 | 2.137 (1.660 - 2.752) | | | |
|  |  | | | | | | |

**Table S52. Coronary atherosclerosis**

|  | | β-coefficient | p-value | Odds Ratio  (95% confidence interval) | | |  |
| --- | --- | --- | --- | --- | --- | --- | --- |
| Intercept | | -8.4473 | <.0001 |  |  |  | |
| Age | | 0.0462 | <.0001 | 1.047 (1.035 - 1.060) | | | |
| Emergency admission | | 0.849 | <.0001 | 2.337 (1.797 - 3.040) | | | |
| Operation | | -1.1604 | <.0001 | 0.313 (0.241 - 0.408) | | | |
| Hypertension, uncomplicated | | -0.6589 | <.0001 | 0.517 (0.403 - 0.664) | | | |
| Other neurological disorders | | 0.6566 | 0.0087 | 1.928 (1.181 - 3.149) | | | |
| Chronic pulmonary disease | | 1.0463 | <.0001 | 2.847 (2.175 - 3.727) | | | |
| Diabetes, uncomplicated | | 0.3543 | 0.0078 | 1.425 (1.098 - 1.850) | | | |
| Renal failure | | 1.7134 | <.0001 | 5.548 (4.076 - 7.551) | | | |
| Peptic ulcer disease excluding bleeding | | -0.487 | 0.0215 | 0.614 (0.406 - 0.931) | | | |
| Coagulopathy | | 1.7457 | <.0001 | 5.730 (3.891 - 8.438) | | | |
| Weight loss | | 1.3376 | <.0001 | 3.810 (2.375 - 6.111) | | | |
| Fluid and electrolyte disorders | | 1.0238 | <.0001 | 2.784 (2.028 - 3.821) | | | |
| Deficiency anemia | | 0.3743 | 0.0283 | 1.454 (1.041 - 2.032) | | | |
|  |  | | | | | | |
